# Ubr1-induced selective endophagy/autophagy protects against the endosomal and Ca^2+^-induced proteostasis disease stress

**DOI:** 10.1007/s00018-022-04191-8

**Published:** 2022-03-01

**Authors:** Ben B. Wang, Haijin Xu, Sandra Isenmann, Cheng Huang, Xabier Elorza-Vidal, Grigori Y. Rychkov, Raúl Estévez, Ralf B. Schittenhelm, Gergely L. Lukacs, Pirjo M. Apaja

**Affiliations:** 1 Lifelong Health, Organelle Proteostasis Diseases, South Australian Health and Medical Research Institute (SAHMRI), 5000 North Terrace, Adelaide, SA 5000 Australia; 2 EMBL Australia, Adelaide, South Australia 5000 Australia; 3 Department of Molecular and Biomedical Sciences, University of Adelaide, Adelaide, SA 5005 Australia; 4 College of Public Health and Medicine, Molecular Biosciences Theme, Flinders University, Bedford Park, SA 5042 Australia; 5 School of Medicine, University of Adelaide, Adelaide, SA 5005 Australia; 6 Department of Physiology and Cell Information Systems, McGill University, 3655 Promenade Sir-William-Osler, Montréal, QC H3G 1Y6 Canada; 7 Department of Biochemistry, McGill University, Montréal, QC H3G 1Y6 Canada; 8 Unitat de Fisiologia, Departament de Ciències Fisiològiques, IDIBELL-Institute of Neurosciences, L’Hospitalet de Llobregat, Universitat de Barcelona, Barcelona, Spain; 9 Centro de Investigación en Red de Enfermedades Raras (CIBERER), ISCIII, Madrid, Spain; 10 Monash Biomedical Proteomics Facility, Department of Biochemistry, Monash Biomedicine Discovery Institute, Monash University, Clayton, VIC 3800 Australia

**Keywords:** Regeneration, Protein homeostasis network, Reprogramming, Protein stability, Stress response, Lysosome

## Abstract

The cellular defense mechanisms against cumulative endo-lysosomal stress remain incompletely understood. Here, we identify Ubr1 as a protein quality control (QC) E3 ubiquitin-ligase that counteracts proteostasis stresses by facilitating endosomal cargo-selective autophagy for lysosomal degradation. Astrocyte regulatory cluster membrane protein MLC1 mutations cause endosomal compartment stress by fusion and enlargement. Partial lysosomal clearance of mutant endosomal MLC1 is accomplished by the endosomal QC ubiquitin ligases, CHIP and Ubr1 via ESCRT-dependent route. As a consequence of the endosomal stress, a supportive QC mechanism, dependent on both Ubr1 and SQSTM1/p62 activities, targets ubiquitinated and arginylated MLC1 mutants for selective endosomal autophagy (endophagy). This QC pathway is also activated for arginylated Ubr1-SQSTM1/p62 autophagy cargoes during cytosolic Ca^2+^-assault. Conversely, the loss of Ubr1 and/or arginylation elicited endosomal compartment stress. These findings underscore the critical housekeeping role of Ubr1 and arginylation-dependent endophagy/autophagy during endo-lysosomal proteostasis perturbations and suggest a link of Ubr1 to Ca^2+^ homeostasis and proteins implicated in various diseases including cancers and brain disorders.

**Supplementary Information:**

The online version contains supplementary material available at 10.1007/s00018-022-04191-8.

## Introduction

Disease-causing mutations, as well as perturbations in intracellular Ca^2+^ homeostasis, may result in protein misfolding and/or accumulation in the cytosol or membrane organelles, mitochondrial oxidative damage [1], the endoplasmic reticulum (ER) stress or lysosomal swelling and exocytosis [2] that have been linked to numerous Mendelian disorders, neurodegenerative diseases, and cancers. To protect protein homeostasis (proteostasis), cells have developed spatial or organelle-specific protein quality control (PQC) mechanisms [3]. At the ER, QC mechanisms can recognize various degradation signals (degrons) in the luminal, transmembrane or cytosolic segments of misfolded membrane proteins as part of the ER-associated degradation (ERAD) and ER-phagy pathway [4, 5]. The cytosolic PQC preferentially relies on the coordinated actions of molecular chaperones, ubiquitin (Ub) conjugation machinery, proteasomes (Ub-proteasome systems) and chaperone-mediated autophagy [3].

Conformationally defective cell surface membrane proteins generated either in situ or following their escape from the ER QC are recognized by the peripheral PQC machinery. Increased ubiquitin conjugation (ubiquitination) of non-native plasma membrane (PM) proteins can signal their internalization, as well as ESCRT (endosomal sorting complex required for transport)-dependent targeting to the multivesicular body (MVB) and lysosomal degradation [6–10]. The peripheral PQC has a critical role in maintaining native protein composition and intra- and inter-organelles membrane dynamics between Golgi, the plasma membrane (PM)- and endo-lysosomal compartments, even at the cost of exacerbating the loss-of-functional phenotype of misfolded but partially functional mutants (e.g [6, 7, 11–13]).

The peripheral PQC is less studied than its ER counterpart, and only three QC E3 Ub-ligases have been implicated in the clearance of non-native PM and endosomal membrane proteins for lysosomal proteolysis. The C-terminus of Hsc70-Interacting Protein (CHIP), Nedd-4 (and its yeast homologues Rsp5), as well as the RFFL, represent both chaperone-dependent and -independent peripheral PQC mechanisms [6, 7, 9, 11, 14].

Additional post-translational modifications by sumoylation, phosphorylation or arginylation with ubiquitination may modulate PQC signaling in a substrate-specific manner [15–18]. From these, arginylation has a role outside QC as N-terminal degron in fast proteasomal degradation of N-recognin type proteins [19]. It has been shown that N-terminal arginylation of BiP (HSPA5, GRP78) released from the ER and cytosolic misfolded soluble proteins can be cleared by autophagy upon proteasomal inhibition or cytokine-induced oxidative stress [20–23]. In the current study, we identified a previously unrecognized role for N-recognins in cargo-selective endosomal autophagy (or endophagy) PQC, which is distinct from Ubr1 presently assigned function in the N-degron pathway [6, 7, 9, 11, 14, 19] and misfolded protein degradation from the cytosol and ER [24–27].

The formation of phagophores, the newly formed membranes that engulf autophagy cargo, has been demonstrated at Rab11 + early endosomes [28]. Selective cargo recognition and clearance mechanisms of misfolded endosomal membrane proteins by autophagy and its protective role against endosomal stress remain undefined. To gain mechanistic insights into these processes, we examined the endosomal PQC activity using a disease-associated regulatory membrane protein, megalencephalic leukoencephalopathy with subcortical cyst 1 (MLC1), as well as aspects of PQC of endosomal compartment during Ca^2+^-stress.

MLC1 is the regulatory constituent of the PM macromolecular signaling cluster, which is pertinent for astrocytes homeostasis, motility/morphology [29–31], inflammatory responses [32], signaling and regulation of the brain extracellular space ion homeostasis [33–35]. The integral membrane protein constituents of the cluster are; Na^+^/K^+^-ATPase, TRPV4 cation and ClC-2 chloride channels, EGF receptor and the GlialCAM adhesion molecules [36–39]. GlialCAM is required for the cell surface targeting and stability of MLC1 [13]. The cell surface diffusional partitioning and endosomal dynamics of the signaling cluster, as well as the activity of ClC-2 [38], gap junctions [31, 34] and Na^+^-K^+^-ATPase [40, 41] are regulated by the MLC1/GlialCAM [13]. Disease mutations in MLC1 or GlialCAM and/or altered expression or compromised PM tethering of the cluster result in MLC1-dependent loss of endo-lysosomal organellar identity and impaired cargo such as ClC-2 sorting [13]. Thus, MLC1 has far-reaching consequences on astrocytic and neuronal proteostasis with an incompletely understood molecular mechanism.

Here we show that Ubr1 is a key player of the PQC by protecting the endosomal compartment proteostasis against cumulative biological stresses. We found that disease-causing MLC1 variants elicit endo-lysosomal and intracellular Ca^2+^-stress that is offset by the E3 Ub-ligases CHIP and Ubr1 activities by two distinct mechanisms. One of them is analogous as previously described ubiquitination- and ESCRT-dependent lysosomal proteolysis of non-native cell surface membrane proteins [6, 7, 9, 11]. In addition, we revealed the operation of an alternative, previously unidentified triage mechanism, which entails Ubr1- and SQSTM1/p62-dependent selective endosomal autophagy (endophagy) that are responsible for the clearance of ubiquitinated and arginylated misfolded MLC1 and other clients during Ca^2+^-stress. We propose a role of the Ubr1/SQSTM1/p62-axis in the endosomal PQC mechanism protecting against cytosolic Ca^2+^-stress and endosomal accumulation of misfolded cargoes that may play a role in proteostasis diseases.

## Results

### Mutant MLC1s are targeted for ubiquitination

**Fig. 1:**
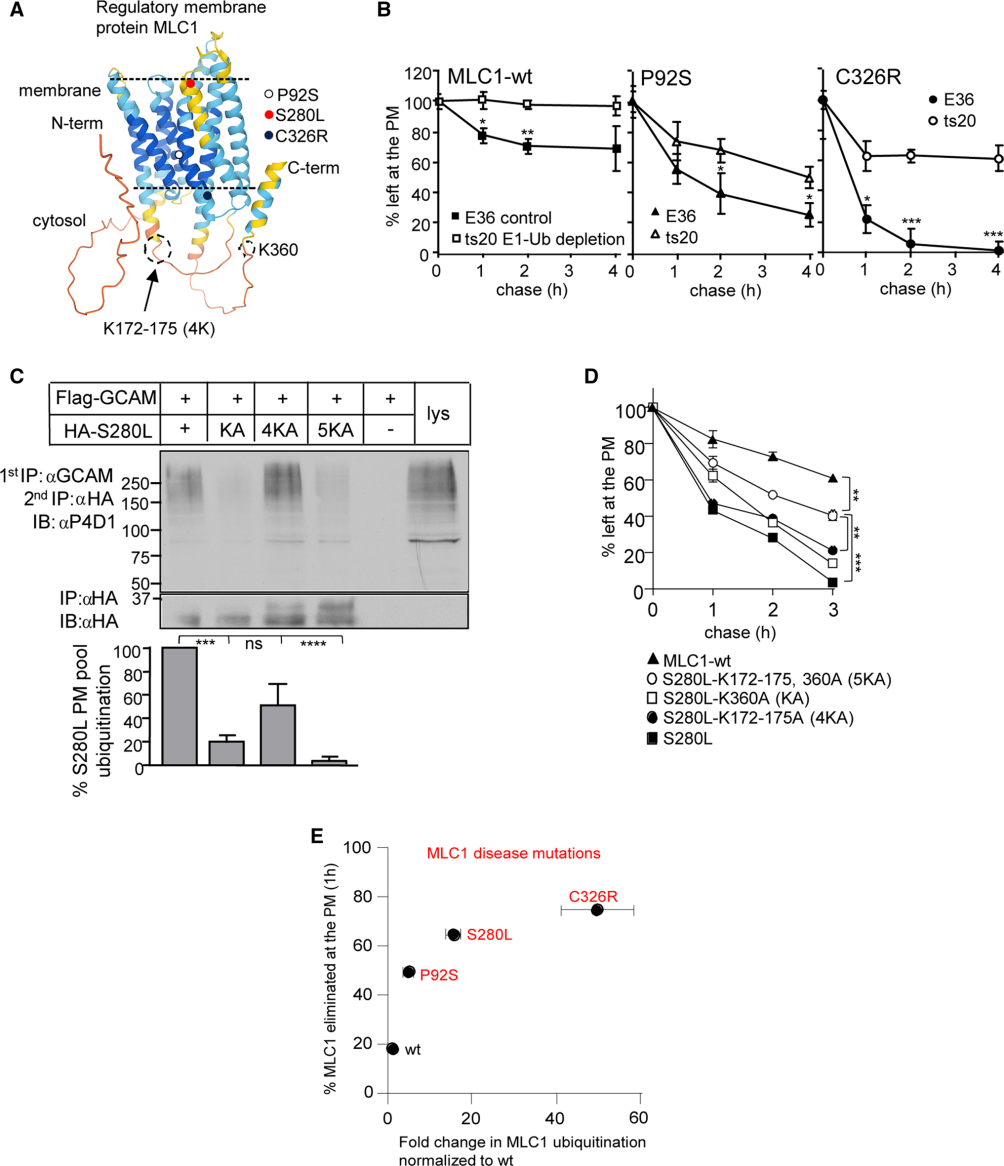
Astrocyte membrane regulatory cluster MLC1 disease variants are ubiquitinated at the PM-endosomal compartment. **A** Predicted regulator membrane protein MLC1 Alphafold [42] structure with indicated disease mutations (e.g. P92S, S280L and C326R) used in this work and ubiquitin lysine (K) acceptor sites used in **C, D**. **B** Lack of the temperature-sensitive (ts) E1 ubiquitin (Ub)-activating enzyme decreased the PM turnover of MLC1 variants. MLC1-wt and disease-associated mutations P92S and C326R were transiently expressed in E36 control and ts20 cells and the E1 was inactivated at 40 ºC for 3 h to remove Ub-conjugation. Live-cell surface (cs)-ELISA for HA-MLC1 and chase for indicated times were used to measure turnover as described in “Methods”. **C** Ub-acceptor lysine mutations decrease the PM-endosomal ubiquitination of misfolded MLC1-S280L. The MLC1/GlialCAM complex was first cell surface immunoprecipitated (cs-IP) using GlialCAM Ab and after the denaturation of complex, a second IP followed with anti-HA Ab to isolate MLC1s. Flag-GlialCAM was transiently expressed in stable inducible HA-MLC1 HeLa [37, 38]. Ubiquitin acceptor Lys residues were mutated (KA = K360, 4KA = K172-175, and 5KA = KA + 4KA, see also **A**). The ubiquitination was normalized to the MLC1-S280L amount (100%) at the PM. GCAM, GlialCAM; lys, lysate; α, anti. **D** Ub-acceptor lysine mutations decrease the PM turnover of MLC1-S280L measured using cs-ELISA as in **A**. **E** Correlation of MLC1 disease variants (P92S, S280L, C326R) PM elimination (%) [13] to the relative fold-change ubiquitination at the PM-endosomes (Fig. S1F, 3E). Means ± SEM, *n* ≥ 3. *P* value: *ns* non-significant, * < 0.05, ** < 0.01, *** < 0.001 **** < 0.0001

GlialCAM is required to chaperone MLC1 folding/assembly into a signaling cluster in the ER and subsequent accumulation at cell surface [13]. Disease-causing mutations in MLC1 or GlialCAM accelerate the cellular and PM turnover of MLC1 variants and result in an enlarged endosomal compartment, an indication of organellar stress [13]. Here we characterized the cell surface degradation mechanism of MLC1 mutations that display progressively increasing stability defect (P92S:mild, S280L:moderate, C326R:severe) [13] in their PM turnovers and expression (Fig. 1A [42]), using doxycycline-inducible HeLa and astrocytic U251N cells, immunoprecipitations (IP) and live-cell surface (cs)-ELISA for the PM-endosomal kinetics (^13, 37, 38^, Methods).

The exogenous MLC1-wt expression level was comparable to that of the endogenous MLC1 in U251N cells (Fig. S1A). To examine the role of ubiquitination in the post-Golgi turnover of MLC1 variants, we expressed MLC1-P92S in ts20 cells, harboring the thermolabile E1 Ub-activating enzyme. Inactivation of the E1 enzyme at 40 °C (Fig. S1B) not only prevented the MLC1-P92S ubiquitination (Fig. S1C) but also decreased its PM turnover, reflected by the three-fold prolonged half-life (T_1/2_) from ~ 1.5 h to ~ 4 h, approaching that of the wt (Fig. 1B). This observation is in line with our conclusion that ubiquitination of MLC1-P92S in post-ER compartments can accelerate the mutant PM turnover. In support, P92S endosomal internalization was also inhibited by > 50% in ts20 cells than in control E36-wt cells and ubiquitination serve as an internalization signal (Fig. S1D). The more pronounced steady-state PM expression defect [37] in MLC1-C326R was accompanied by its accelerated even faster turnover (T_1/2_ ~ 0.5 h), which was also inhibited in ts20 cells (Fig. 1B). These results indicate that the significantly reduced steady-state expression of MLC1 mutants is attributed to their accelerated turnover in post-Golgi compartments. Further, proteasomal inhibition by MG132 (2 h) unmasked increased P92S polyubiquitination relative to wt (Fig. S1E) indicating that the proteasome-dependent proteolysis at the ER partly contributes to mutants reduced steady-state expression in post-ER compartments.

To selectively isolate the PM-resident MLC1, we used cell surface immunoprecipitation (cs-IP), taking advantage of the MLC1 association with GlialCAM [13, 37] at the PM and the antibody (Ab) that recognizes the extracellular domain of GlialCAM. The affinity-purified cell surface MLC1/GlialCAM complex was then denatured and MLC1 was IP-ed to probe for its Ub-conjugation with anti-Ub Ab immunoblotting. The ubiquitination of the PM pools of the mild P92S was increased by ~ 5 fold and the severe C326R mutation by ~ 40-fold relative to native MLC1-wt (Fig. S1F) in post-ER compartments.

To assess whether enhanced ubiquitination is an absolute requirement for the mutant accelerated PM turnover, the previously identified ubiquitin acceptor lysine (K) residues [43] were mutated in MLC1-S280L displaying a moderate turnover defect. Mutagenesis of K360 in the C-terminal tail and the K172-175 cluster (4 K) in the cytosolic loop two decreased the MLC1-S280L ubiquitination (Fig. 1A, C) and turnover (Fig. 1D) at the PM. Although the combination of these lysine mutations (5KA) further decreased the mutant PM ubiquitination, the S280L-5KA variant retained a two-fold faster turnover (T_1/2_ ~ 2 h) than MLC1-wt (T_1/2_ > 4 h, Fig. 1D), suggesting that additional mechanism(s) is likely involved in the mutants clearance from the PM.

In summary (Fig. 1E), the PM ubiquitination propensity of the mutants correlated with their accelerated PM turnover rate. The PM half-lives (T_1/2_) of MLC1 variants were reduced by 2.6-to-8 fold pending on the mutation severity (measured in [13]: wt > 4 h, P92S ~ 1.5 h, S280L ~ 1 h, and C326R ~ 0.5 h) accompanied by a 10–50-fold increase in their relative PM ubiquitination against native MLC1-wt (Fig.S1F, 3E). Notably, the PM turnover of MLC1 variants was comparable in different cell types [13, 37, 38].

### MLC1 mutations perturb cytosolic Ca^2+^ homeostasis and stimulate lysosomal exocytosis

**Fig. 2:**
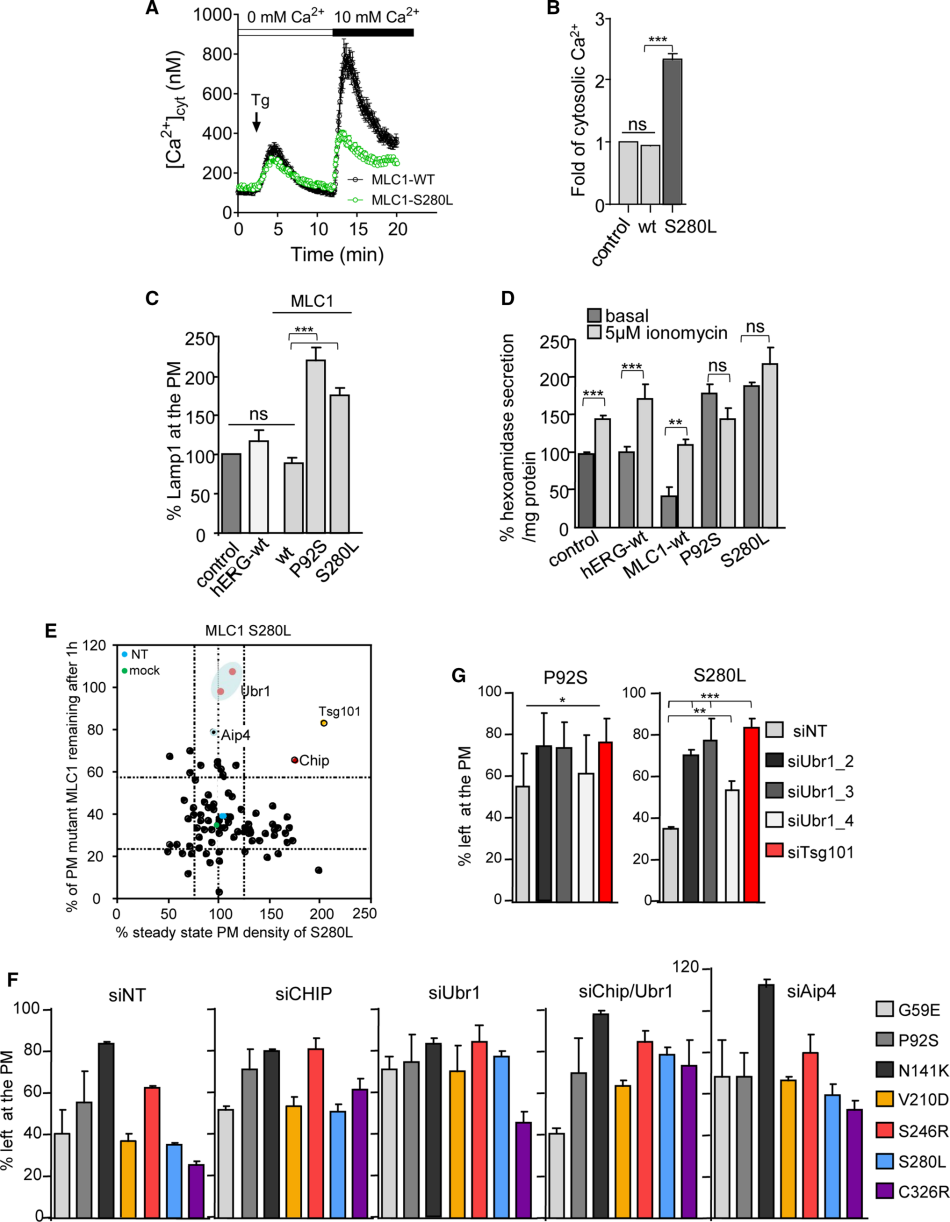
Screening post-Golgi and/or Ca^2+^-stress directed E3 ubiquitin QC ligases. **A** Comparison of extracellular Ca^2+^ uptake across the PM (height of the peak) between HA-MLC1-wt and S280L expressing stable inducible U251N cells following the activation of store-operated Ca^2+^-channels. Fura-2 was loaded in a Ca^2+^-free solution into live cells followed by thapsigargin (Tg) to induce an increase in the cytosolic [Ca^2+^]. The extracellular 10 mM [Ca^2+^] was added to activate Ca^2+^ entry across the PM through Ca^2+^-permeable channels. *n* = 80–100 cells for each trace. **B** MLC1 mutations cause basal cytosolic Ca^2+^ increase. Basal steady-state intracellular Ca^2+^-fold difference between control, MLC1-wt and S280L was measured using Calbryte520 in stable inducible U251N cells. Calbryte520 was loaded to live cells and measured using a fluorescence microplate reader and the signal was normalized to the number of cells. **C** Basal Lamp1 expression at the PM indicating intracellular Ca^2+^-increase was measured using cs-ELISA (as in Fig. 1B) in MLC1-wt, P92S, S280L stable inducible U251N cells and control and hERG channel expressing cells. **D** Release of lysosomal enzyme β-hexosaminidase was measured using fluorometric assay from the medium of mock and 5 µM ionomycin treated cells as in **C**. **E** Phenotypic siRNA screen of candidate QC E3-ligases for the PM-endosomal clearance of MLC1-S280L causing intracellular Ca^2+^ overload. The PM expression and stability of HA-MLC1-S280L were measured using cs-ELISA (as in Fig. 1B) expressed in stable inducible HeLa. ESCRT-I protein Tsg101 (siTsg101) was used as a positive and siNT as a negative control. The potential PM/endocytic candidates CHIP, Aip4 and Ubr1 are indicated and lines of 3 ± SD. **F** Cells were depleted with siRNA for QC E3-ligases CHIP, Ubr1, CHIP/Ubr1 and Aip4 and control siNT and the PM turnover of disease-associated MLC1 variants [13] were measured using cs-ELISA (as in Fig. 1B). **G** Ubr1 depletion decreases the PM turnover of misfolded MLC1. Cells were depleted using three different Ubr1 siRNA target sequences. siNT served as a negative and siTsg101 as a positive control. Cs-ELISA was used to measure the PM turnover after the 1 h chase (as in Fig. 1B). Means ± SEM, *n* ≥ 3. *P *value: *ns* non-significant, * < 0.05, ** < 0.01, *** < 0.001

The cytoplasmic [Ca^2+^] changes are appreciated as a proteostasis modulator. MLC1 mutations cause endosomal compartment fusions, inhibition of fission and/or intraluminal vesicle budding without altering the lysosomal proton permeability [13], which could relate to perturbations in the cytosolic [Ca^2+^]. The cytosolic [Ca^2+^]-transients upon activation of the store-operated Ca^2+^ influx from the extracellular space were examined using Fura-2 Ca^2+^-sensitive dye [44] in MLC1-wt and S280L U251N cells. Cells expressing MLC1-S280L exhibited diminished Ca^2+^-entry through the PM store-operated Ca^2+^-channels in response to the depletion of intracellular Ca^2+^-stores by thapsigargin (Tg) relative to MLC1-wt cells (Fig. 2A). This could explain the ~ 2.5-fold elevation of the resting cytosolic [Ca^2+^] in MLC1-S280L relative to MLC1-wt expressing cells, measured using fluorescent indicator Calbryte520 (Fig. 2B).

As lysosomal exocytosis is promoted by the cytosolic [Ca^2+^] elevation, we monitored the lysosomal associated membrane protein Lamp1 exocytosis to the PM [45] and the constitutive and ionomycin-stimulated (5 µM) hexosaminidase secretion [46]. The constitutively increased Lamp1 at the PM, hexosaminidase secretion and the loss of ionomycin-triggered hexosaminidase exocytosis in MLC1-P92S and S280L cells were consistent with their elevated cytosolic [Ca^2+^] level and the organellar stress associated with endosomal compartment enlargement [13] (Fig. 2C, D).

### Identification of QC E3-ligases in post-Golgi compartments

The mechanism(s) of how endosomal compartments attenuate proteostasis stress is not understood. To identify Ub E3-ligases that may be responsible for signaling the disposal of non-native endosomal membrane proteins and/or protect endosomal homeostasis during Ca^2+^-perturbations, we performed a cell-based phenotypic siRNA sub-library screen against candidate E3-ligases that may be engaged in PQC. The objective was to identify E3-ligases that can impede the accelerated PM-endosomal turnover of the MLC1-S280L.

The PM turnover of MLC1-S280L was measured after one hour chase at 37 °C using cs-ELISA in siRNA-transfected HeLa cells lacking endogenous MLC1. Depletion of targets that delayed the mutant turnover more than three standard errors relative to that of non-target siRNA (siNT) controls were considered as positive hits (Fig. 2E). siRNA against TSG101, which is indispensable for ESCRT-dependent lysosomal degradation of ubiquitinated misfolded PM proteins, was included as a positive control [6, 7, 9, 47].

Downregulation of CHIP, AIP4, and Ubr1 significantly delayed the MLC1-S280L turnover (Fig. 2E and Fig.S2A). CHIP is known to be involved in PQC at multiple organelles [6, 7, 9] while AIP4 (Itch) ubiquitinates cytosolic toxic misfolded proteins [48] and a subset of native endosomal receptors and adaptor proteins [49]. The N-recognin function of Ubr1 (Ubiquitin Protein Ligase E3 Component N-Recognin 1) is to target N-degrons in short-lived cytosolic and unfolded proteasomal substrates. In addition, separately as a PQC-ligase, Ubr1 is involved in the clearance of misfolded yeast ER and mitochondrial membrane protein [19, 26, 27, 50]. As siAIP4 altered the endo-lysosomal transfer kinetics of constitutively ubiquitinated and recycling model cargoes (CD4t-Ub_n_, CD4cc-UbRΔG_4_) and the transferrin receptor (TfR) [51–53] (Fig. S2C-D) suggesting alternative functions to PQC, we focused our studies on CHIP and Ubr1.

Depletion of Ubr1 (siUbr1) and CHIP (siCHIP) decreased the PM turnover of six MLC1 disease-causing mutations in the transmembrane/cytosolic segments without an additive effect or influencing the wt and the native-like N141K mutant (Figs. 1A, 2F, S2B). The specificity of siUbr1 to diminish the fast PM turnover of P92S and S280L was confirmed using three different siUbr1 (Fig. 2G). Ubr1 depletion did not influence constitute recycling of TfR or the lysosomal targeting of the Ub-dependent (CD4t-Ub_n_, CD4cc-UbRΔG_4_) and Ub-independent (CD4t-LAMP) model transmembrane cargoes [51–53] (Fig.S2C-D). These results suggest that CHIP and Ubr1 catalyzed ubiquitination can discriminate between misfolded and native MLC1. Furthermore, they have an undetectable effect on the ubiquitinated cargo sorting by the ESCRT machinery in our cellular model.

**Fig. 3:**
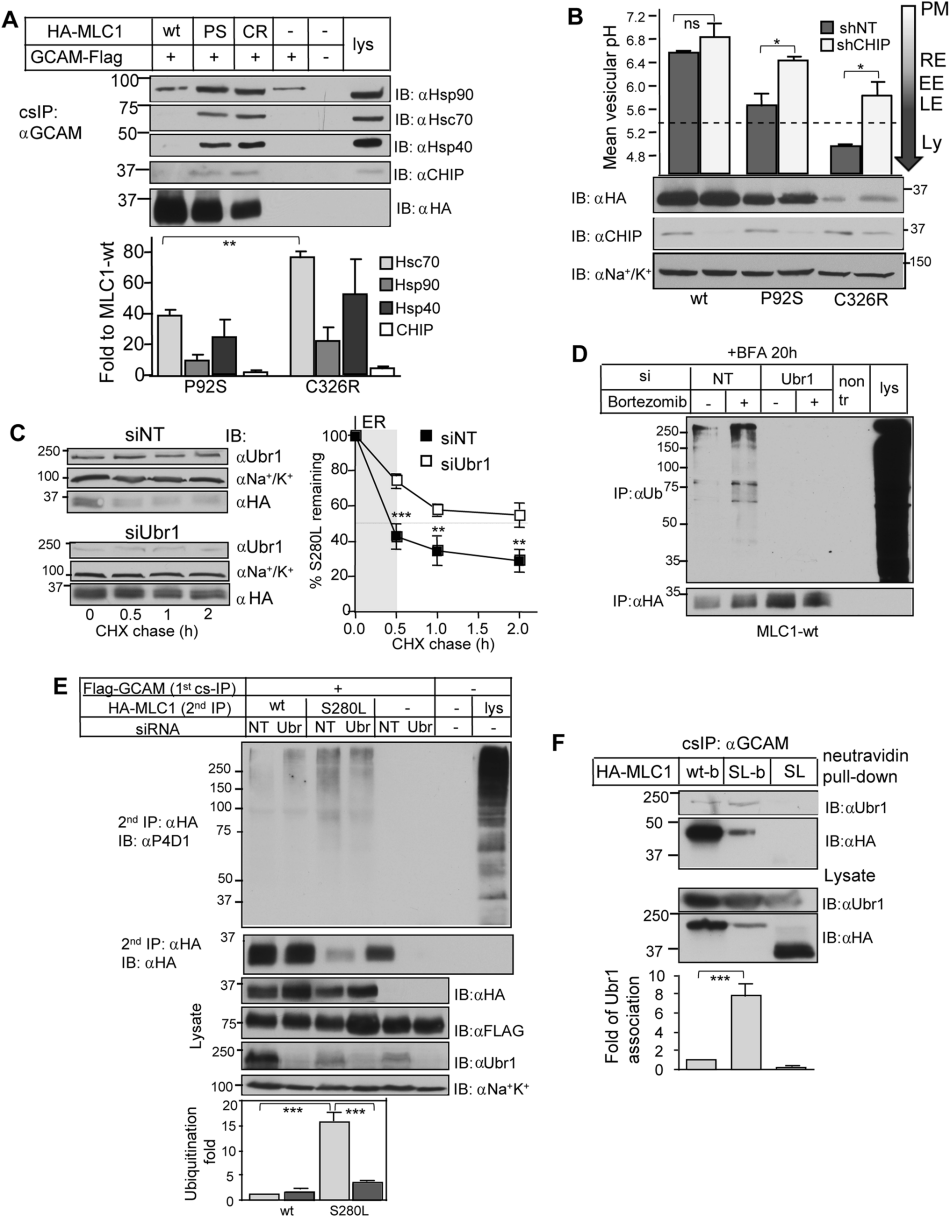
Disease-causing MLC1 variants are substrates for Ubr1. **A** Interaction of molecular chaperones and E3 QC-ligase CHIP with misfolded MLC1 was monitored at the PM-endosomes. The MLC1/GlialCAM complex was cell surface immunoprecipitated (cs-IP) using GlialCAM Ab and protein interactions analyzed using Western blotting. Flag-GlialCAM was transiently expressed in stable inducible HA-MLC1 HeLa. The chaperone/CHIP amount was normalized to MLC1 expression at the PM. GCAM, GlialCAM; lys, lysate; α, anti. **B** Mean single vesicular pH was measured from MLC1 containing endosomes using live-cell microscopy after 1 h chase in cells depleted for CHIP with a short hairpin (sh) or shNT. The PM MLC1 was labeled with a pH-sensitive fluorophore and endosomal pH was measured as in Methods. The average weighted mean was calculated from at least three independent experiments, and > 250 vesicles from ~ 25–50 cells/ experiment were analyzed. Cells were as in A. Tha PM, recycling endosome (RE) pH 6.3–6.2, early endosome (EE) and late endosomes (LE) /lysosomes (L) < 5.5 are indicated. **C** The effect of Ubr1 depletion on MLC1-S280L total protein turnover was measured using CHX-chase and Western blot analysis. Control cells were treated with siNT. Cells were as in A. **D** ERAD and Ubr1 were monitored in siRNA-treated cells for Ubr1 or siNT. BFA was added for 20 h to prohibit MLC1-wt export from the ER. Proteasomal degradation was inhibited with Bortezomib for 2 h to accumulate ubiquitinated non-native MLC1. The ubiquitination signal was monitored after denaturation and IP for MLC1. Cells were as in A. **E** The effect of Ubr1 depletion on PM-endosomal ubiquitination of native MLC1-wt and misfolded S280L was measured using cs-ubiquitination assay and quantification as in Fig. 1C. Cells were as in A. **F** Proximity-dependent biotin protein–protein interaction assay between MLC1 and Ubr1 was probed in cells transiently expressing HA-MLC1-wt-Bir* (wt-b), S280L- Bir* (SL-b) or S280L (SL). Cells were treated with 50 µM biotin for 20 h and MLC1 was isolated using cs-IP (as in A). Biotinylated Ubr1 was recovered on neutravidin beads and analyzed using Western blotting. The biotinylated Ubr1 amount was normalized for the PM amount of MLC1. *GCAM* GlialCAM, *lys* lysate, *α* anti. Means ± SEM, *n* ≥ 3. *P *value: *ns* non-significant, * < 0.05, ** < 0.01, *** < 0.001

CHIP recruitment to non-native polypeptides is facilitated by molecular chaperones and co-chaperones [54–56]. Immunoblotting of the cs-IPed MLC1/GlialCAM complex revealed ~ 40–80-fold increased association of Hsc70, Hsp90, and Hsp40 (DNAJB1) with the severe MLC1-C326R and ~ 50% less with milder MLC1-P92S relative to the MLC1-wt, paralleling the severity of their PM stability defects. DNAJB1 type Hsp40 recruits misfolded substrates to Hsp70, as well as targets a range of human Hsp70-based disaggregase clients [57]. CHIP association was increased by ~ 2–5 fold in mutants relative to the MLC1-wt (Fig. 3A). CHIP siRNA depletion attenuated the MLC1-P92S PM turnover by ~ 66% and internalization by ~ 50%, which was reversed by CHIP-wt overexpression (Fig. S3A–C). The amount of chaperone/CHIP recruitment correlated with the severity of misfolded PM/endosomal MLC1 in the cluster, although the contribution of other members (e.g. GlialCAM or ClC2) in the cluster could not be ruled out [37, 38].

If endosomal ubiquitination and ESCRT-dependent lysosomal targeting of non-native MLC1s are influenced by the dynamics of Ub-conjugation and deubiquitination [52, 58], CHIP depletion should delay their endosomal-lysosomal transfer kinetics. To determine the transport kinetics, we followed MLC1-vesicle endocytosis using pH-sensitive single vesicle fluorescence microscopy analysis in live cells [6, 7, 59]. The endo-lysosomal localization was based on the progressive acidification of the endo-lysosomal vesicular pH (pH_v_), ranging from pH ~ 7.2 to 4.5 [6, 7, 59–61]. MLC1-wt was confined to recycling endosomes (pH_v_ 6.3 ± 0.2) after 1 h endocytosis, while the P92S (pH_v_ 5.65 ± 0.3) and C326R (pH_v_ 5.0 ± 0.04) were targeted to the MVB (late endosomes)/lysosomes based on their more acidic pH_v_ (Fig. 3B). The mutant delivery to MVB/lysosomes was delayed by depletion of CHIP or ESCRT-0 and -I constituents STAM, Hrs or TSG101 (Figs. 3B and S3D, E). These data indicate that the accelerated PM/endosomal clearance of a fraction of misfolded MLC1 is mediated by the ubiquitination- and ESCRT-dependent PQC.

### ER and post-ER ubiquitination of MLC1 by Ubr1

Lack of CHIP caused partial inhibition of endo-lysosomal transfer kinetics of MLC1 variants (Fig. 3B). Albeit Ubr1 has been implicated in yeast PQC, independently of its role as N-recognin, toward a small number of misfolded cytosolic, ER, mitochondrial clients [26, 27, 50], Ubr1 in the human ER and endosomal PQC is incompletely understood. We selected the MLC1-S280L with moderate expression and turnover defect for further QC studies (Fig. 1A, E).

To assess the involvement of Ubr1 in cellular turnover of MLC1-S280L, we measured the mutant stability upon translational inhibition with cycloheximide (CHX) and immunoblotting in siUbr1- and siNT-treated cells. The fast initial degradation rate of MLC1-S280L during the first 0.5 h CHX-chase was inhibited by ~ 50% in siUbr1 exposed cells (Fig. 3C), suggesting that Ubr1 contributes to the ER clearance of misfolded MLC1. To address this possibility, the non-native MLC-wt was accumulated in the ER by preventing the ER export with Brefeldin A (BFA) and the ERAD with proteasomal inhibitor Bortezomib. The ubiquitination of the accumulated non-native MLC-wt was reduced by > 75% in siUbr1 treated cells (Fig. 3D). These results suggested that Ubr1-dependent ubiquitination contributes to the unassembled and/or incompletely folded MLC1 targeting for ERAD.

Next, we interrogated Ubr1 capacity for Ub-conjugation at the PM-endosome. MLC1/GlialCAM was isolated using cs-IP followed by a second IP for MLC1 after denaturation to reveal MLC1 ubiquitination with immunoblotting and anti-Ub Ab (Fig. 3E, as in Fig. 1C, S1F). Ubiquitination was normalized to the PM isolated MLC1 amount. Ubr1 depletion decreased the misfolded MLC1-S280L ubiquitination by ~ 75% at the PM, while its PM density was increased by ~ 3 fold (Fig. 3E, *lower panel*) without affecting natively folded MLC1-wt abundance and ubiquitination at the PM, suggesting that Ubr1 is responsible for the misfolded MLC1 ubiquitination at the PM-endosomes.

To demonstrate the physical proximity of Ubr1 and MLC1 at PM-endosomes, misfolded MLC1-S280L was expressed with Flag-Ubr1-wt or catalytically inactive (Ubr1-CI). After mild in vivo cross-linking, S280L/GlialCAM was isolated using cs-IP without denaturation. Ubr1-wt reduced, while the Ubr1-CI elevated the MLC1-S280L amount at the PM, however, Ubr1 detection was low.

To improve the detection sensitivity, we measured the MLC1/Ubr1 interaction with the proximity biotinylation [62] technique using MLC1-Bir* chimera as the bait and cs-IP. The endogenous Ubr1 displayed ~ 8 fold higher biotinylation susceptibility by the MLC1-S280L-Bir* than MLC1-wt (Fig. 3F). Jointly, these results uncovered that non-native MLC1 variants represent previously unrecognized PQC clients for Ubr1 in the ER and PM-endosomes.

### Ubr1 functions as a PM-endosomal QC E3-ligase

The subcellular localization of Ubr1 in human cells is now well characterized. Endogenous Ubr1 and heterologously expressed Flag-Ubr1 and mCherry-Ubr1 localizations were evaluated by indirect immunostaining and fluorescence microscopy without the presence of misfolded protein or Ca^2+^-stress. Ubr1 was associated with membrane puncta and partly with the PM (Fig. S4A), phalloidin stained F-actin at endosomal invaginations, as well as clathrin adaptors (AP2) and clathrin light chains (CLC) (Fig. S4B), being consistent with its role at the PM-endosomal compartment. Additionally, Ubr1 was found partly in the nuclei (Fig. S4A) and with the ER-markers KDEL and ERp57, but not with the lysosome marker Lamp1 or autophagosome marker LC3b (Fig. S4B).

**Fig. 4:**
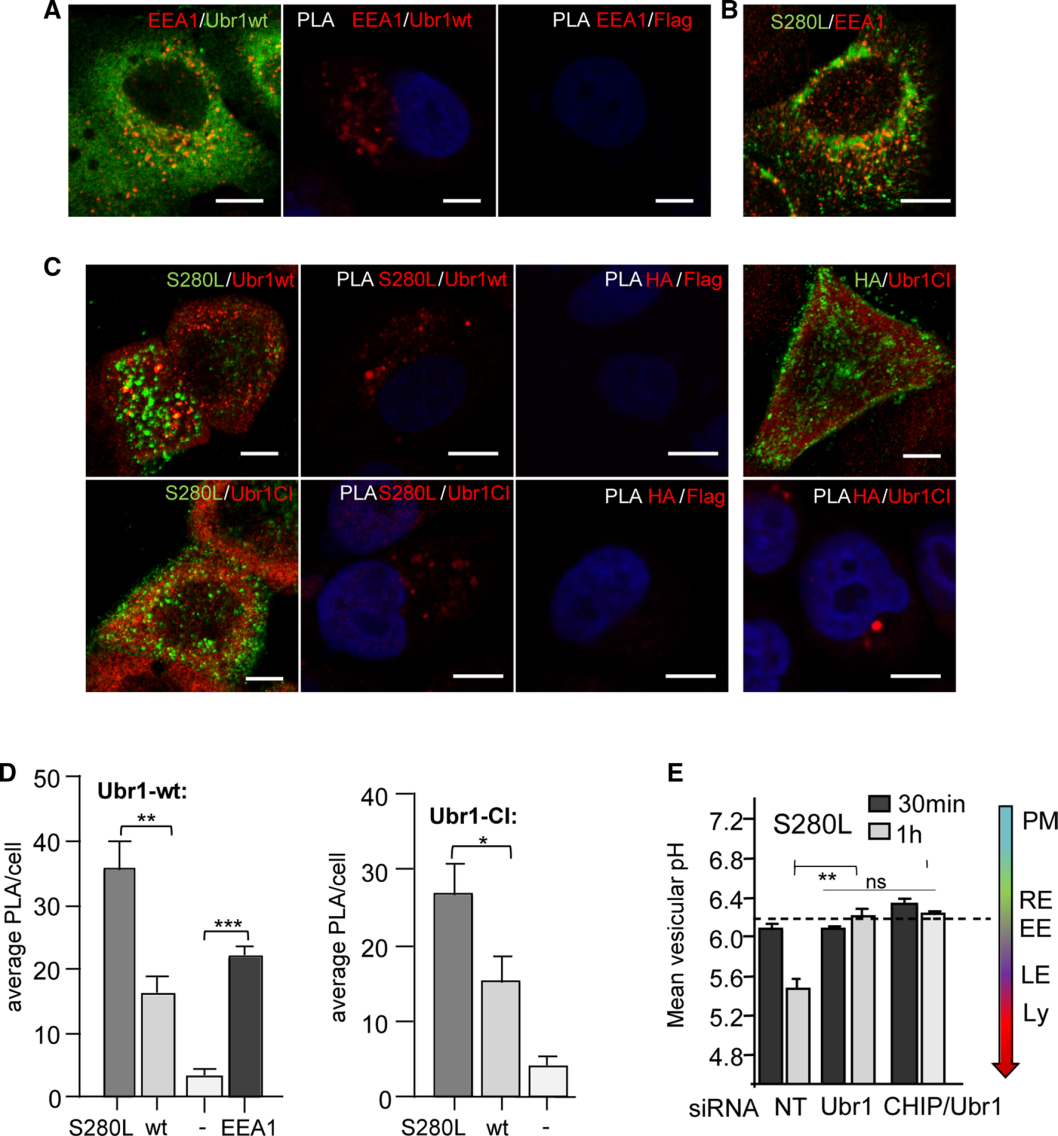
Ubr1 functions as an endosomal QC E3 ubiquitin ligase. **A** Immunofluorescence microscopy for early endosomal antigen EEA1 and Ubr1 interaction was detected using anti-EEA1 and anti-Flag Abs, and protein–protein interaction with PLA was performed between EEA1 and Flag epitopes. Cells without Flag-Ubr1-wt expression were used as a control. Bar 5 µm. **B** Immunofluorescence colocalization of internalized (30 min) endosomal MLC1-S280L was visualized using anti-HA with early endosomal EEA1. **C** Immunofluorescence of endosomal MLC1-S280L or MLC1-wt and Ubr1 interaction was determined for internalized anti-HA after 30 min and stained for Flag-Ubr1-wt or CI using anti-Flag. PLA was performed between HA-Flag epitopes and cells without MLC1 were used as a control. Bar 5 µm. **D** Quantification of mean protein interaction between Flag-Ubr1-wt or CI or control (−) and MLC1-wt, S280L or EEA1 with PLA from > 30 cells. **E** Mean vesicular pH measurement of S280L containing endosomes was determined using live-cell single fluorescence microscopy in cells depleted for CHIP, Ubr1, CHIP/Ubr1 or control siNT. *PM* plasma membrane, *RE* recycling endosome (pH 6.3–6.2), *EE* early endosomes, *LE* late endosome, *Ly* lysosome. Means ± SEM, *p* value: *p < 0.05, **p < 0.01, *** < 0.001

The Flag-Ubr1 colocalization with endocytosed native MLC1-wt or misfolded MLC1-S280L was determined next. Extracellular MLC1 HA-epitope was selectively labeled at the PM and endocytosed for 30 min for immunofluorescence or proximity ligation assay (PLA) [37, 38] to visualize protein–protein interactions. Both Ubr1 and MLC1-S280L colocalized with early endosomal marker EEA1 (Fig. 4A, B). We confirmed the preferential interaction of endosomal misfolded MLC1-S280L with the Ubr1-wt and Ubr1-CI, as well as EEA1 + using the PLA (Fig. 4C, D). The immunolocalization and proximity interaction were substantially enhanced between Ubr1-CI and S280L over the MLC1-wt at the PM proximity without endocytosis, reinforcing the notion that Ubr1 preferentially interacts with misfolded MLC1 at PM-endosomal compartments.

Next, the contribution of Ubr1 on the endosomal sorting of MLC1 was interrogated by monitoring misfolded MLC1-S280L endo-lysosomal trafficking with pH_v_-analysis (as in Fig. 3B) in control and siUbr1-depleted cells after 0.5 and 1 h chase. Ubr1 depletion prevented the delivery of MLC1-S280L from early endosomes (pH_v_ 6.1 ± 0.08) to late endosome/MVB (pH_v_ 5.5 ± 0.02) during the 30–60 min chase period (Fig. 4E). A comparable effect was documented upon CHIP and Ubr1 depletion (Fig. 4E). Notably, Ubr1 depletion did not alter the fluid-phase endocytosis marker dextran lysosomal targeting or the ubiquitinated model substrate CD4t-Ub_n_ (Fig. S4D, see also Fig. S2C-E), ruling out its non-specific effect on ESCRT-dependent ubiquitinated cargo sorting or bulk cargo lysosomal delivery. Jointly, these results strongly suggest that Ubr1 activity is indispensable for the timely delivery of misfolded endosomal MLC1 variants into MVB/lysosomes.

### Ubr1 is activated by proteostasis stress in endosomal compartments

**Fig. 5:**
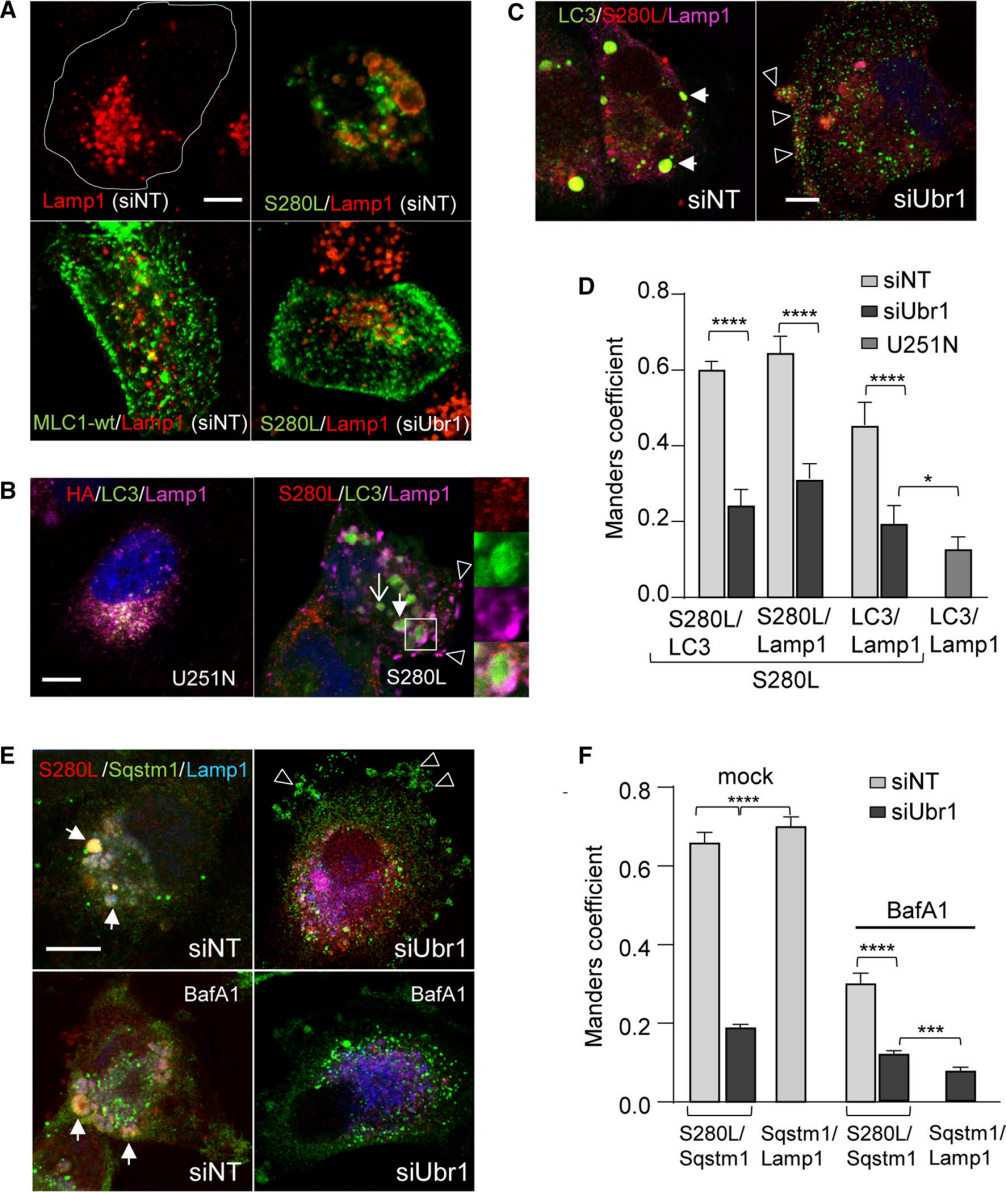
Ubr1 is required for endophagy activation. **A** The effect of Ubr1 expression on the endo-lysosomal pathway morphology was monitored by immunofluorescence microscopy in siRNA-treated cells for Ubr1 or control NT. Endosomal MLC1-S280L or wt was labeled by anti-HA Ab capture for 30 min (37 °C) and visualized by anti-mouse secondary Ab. Lamp1 was used as a lysosomal marker to detect the enlarged lysosomal phenotype. Bar 5 µm. **B** Autophagosome marker LC3b and lysosomal marker Lamp1 were used to identify autolysosomes (arrow, insert) and autophagosomes (open arrow) in MLC1-S280L cells as in A. Arrowhead (empty) points to exocytosed lysosomes at the PM. Bar 5 µm. **C** Endosomal MLC1-S280L and Ubr1 connection to the endosomal stress and formation of autophagosomes/autolysosomes (endophagy) was monitored in Ubr1 depleted (siUbr1) or control siNT cells as in A. LC3b was used as an autophagosome and Lamp1 as a lysosomal marker. The arrow indicates autolysosomes positive for S280L, Lamp1 and LC3b, and the arrowhead points to the PM relocated S280L, Lamp1 and LC3b. Bar 5 µm. **D** Manders' overlap coefficient of indicated autophagy (LC3b) and lysosome (Lamp1) markers. Representative cells are in B–C. *n* > 40 cells. **E** Connection of Ubr1 and endosomal MLC1-S280L to the endosomal stress and recruitment of autophagy scaffold SQSTM1/p62 to phagophores in MLC1-S280L cells was done as in **A**. BafA1 was used to inhibit autophagosome-lysosome fusions, which resulted in the cytosolic dispersion of SQSTM1/p62. The arrow indicates autolysosomes and arrowheads the SQSTM1/p62 positive PM blebs. Bar 5 µm. **F** Manders' overlap coefficient of selective autophagy scaffold SQSTM1/p62 and lysosomes (Lamp1) with HA-MLC1-S280L. Representative cells are in **E**. *n* > 26 cells. Means ± SEM, *p* value: *p < 0.05, ***p < 0.001, ****p < 0.0001

The role of Ubr1 in a cytosolic and ER QC has been demonstrated upon cellular stress in yeast [24, 27]. Given that mutant MLC1 perturbed Ca^2+^-homeostasis (Fig. 2A–D) and endosomal fusions [13], we asked whether stress signaling can attribute to Ubr1 activation to PQC. MLC1-pool was labeled with anti-HA Ab and endocytosed (30 min at 37 °C) and compared to Lamp1 + lysosomes using immunofluorescence microscopy. Only MLC1-S280L but not the MLC1-wt provoked the accumulation of enlarged Lamp1 + late endosomes/lysosomes (Fig. 5A). Importantly, Ubr1 activity/binding was required for the accumulation of the enlarged MLC1-S280L/Lamp1 + late endosomes/lysosomes as Ubr1 depletion prevented this phenomenon (Fig. 5C-D). Remarkably, enlarged Lamp1 + late endosomes/lysosomes were positive for LC3b and MLC1-S280L identifying them as autolysosomes (Fig. 5B–D). A subpopulation of LC3b and MLC1-S280L positive vesicles were Lamp1 negative and likely represent newly formed cargo-selective autophagosomes (or endo-phagosomes) (Fig. 5B open arrow).

**Fig. 6:**
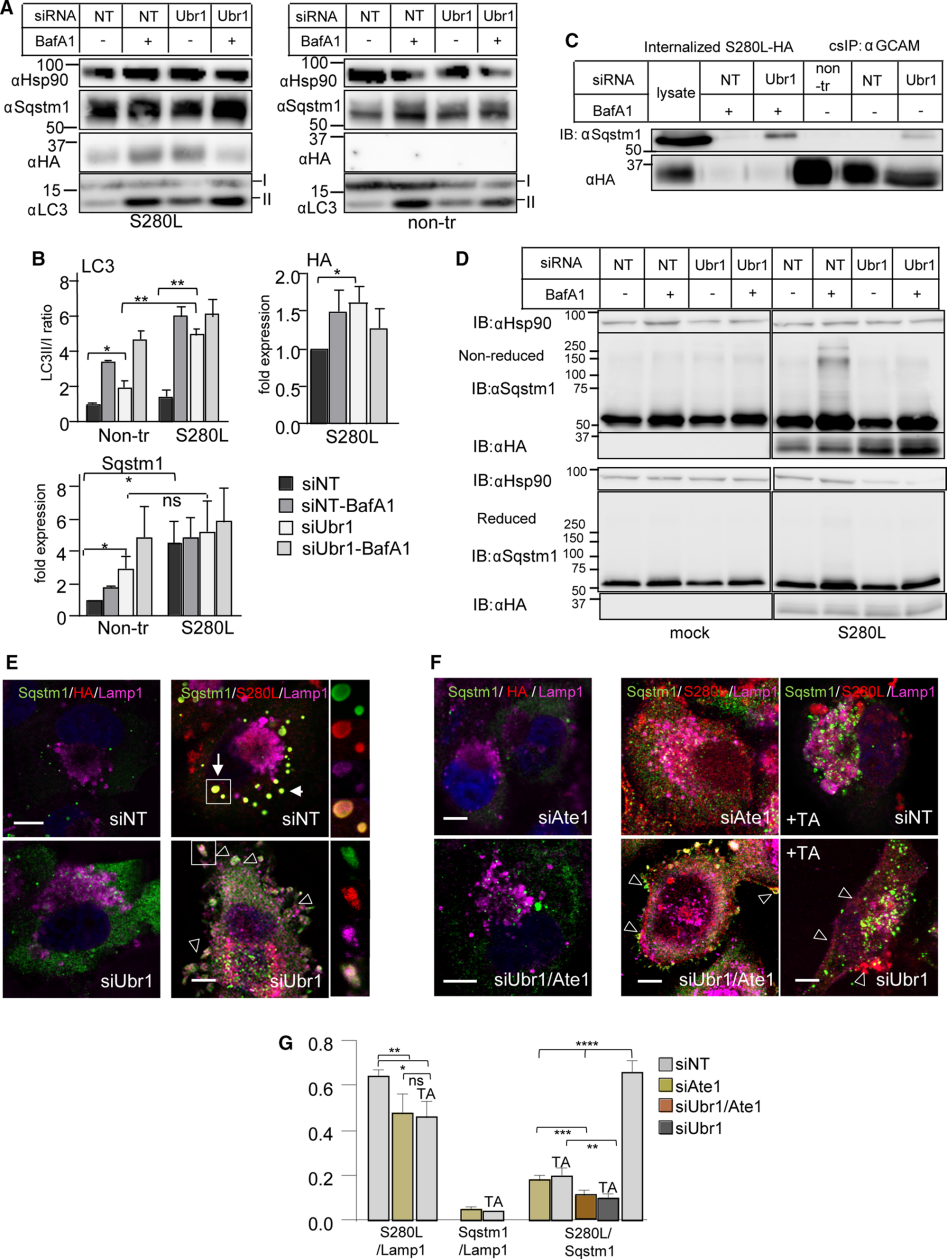
Ubr1 is required as a proteostasis stress QC-ligase for the activation of selective SQSTM1/p62 endophagy. **A** Western blot analysis of cells depleted with siRNA for Ubr1 or NT to measure the expression of SQSTM1/p62 and the formation of mature autophagosomes using lipidated/cytosolic LC3b-II/I ratio in HA-MLC1-S280L and non-expressing U251N cells. BafA1 was used to inhibit autophagosome-lysosome fusions to decrease autophagosome-cargo degradation. Hsp90 was a loading control. **B** Densitometric quantification of MLC1-S280L, SQSTM1/p62 and lipidated/cytosolic LC3b-II/I ratio from **A**. *n* = 3. **C** Protein–protein interaction between autophagy scaffold SQSTM1/p62 and endosomal MLC1-S280L was measured using cs-IP (as in Fig. 3A) for cells treated as in A. **D** Oligomerization of SQSTM1/p62 requires Ubr1 in the presence of MLC1-S280L. Cells treated as in A were analyzed by reducing and non-reducing immunoblotting. **E, F** Immunofluorescence microscopy of Ubr1 and arginylation effect on SQSTM1/p62 recruitment. The endo-lysosomal pathway morphology was monitored in HA-MLC1-S280L or non-expressing U251N cells treated with siUbr1 or siNT. Endosomal MLC1-S280L and control were labeled by anti-HA Ab capture (30 min, 37 °C) and visualized for colocalization with Lamp1 lysosomal marker and the selective autophagy scaffold protein SQSTM1/p62 (as in 5E) in the absence or presence of arginylation by Ate or arginylation inhibitor tannic acid (TA). The arrow indicates autolysosomes and arrowheads lysosomal Lamp1 or SQSTM1/p62 at the PM and in blebs. The insert illustrates S280L containing autophagosome (upper panel) or the PM trapped cargo phagophore (lower panel). Bar 5 µm. **G** Manders' overlap coefficient of selective autophagy scaffold SQSTM1/p62 and lysosomes (Lamp1) with HA-MLC1-S280L. Representative cells are in **E**, **F**. *n* > 29. *non-tr* non-transfected, *α* anti, means ± SEM, *p* values: *p < 0.05, **p < 0.01, ***p < 0.001, ****p < 0.0001

Interaction of Ubr1 with misfolded endosomal MLC1 and the formation of large LC3b and Lamp1/LC3b positive autophagosomes/autolysosomes suggest that Ubr1 has endo-lysosomal stress QC functions. In support, Ubr1 depletion by siRNA abrogated the formation of autolysosomes and their precursors (endosome-derived autophagosome) dispersing LC3b to the cytosol and PM resulting in ~ 3 fold decrease in colocalization of S280L/LC3b and S280L/Lamp1 (Fig. 5C, D). Importantly, formed Lamp1 + autolysosomes were positive for the stress-induced SQSTM1/P62 scaffold that targets ubiquitinated proteins for selective autophagy [63] (Fig. 5E, F). Consistently, Ubr1 depletion prevented the accumulation of SQSTM1/P62 into the forming autophagosomes, dispersed SQSTM1/P62 into the cytosol and endosomal anti-HA labeled MLC1-S280L to low-curvature PM blebs. The colocalization of S280L/SQSTM1 was decreased by ~ 3 fold upon Ubr1 depletion (Fig. 5E, F, also Fig. 6E).

Inhibiting the autophagosome-lysosome fusion by bafilomycin A1 (BafA1) or chloroquine (Chq) resulted in cytosolic dispersion of SQSTM1/p62 and the reduced colocalization of the SQSTM1/p62 with MLC1-S280L, consistent with partial degradation of the mutant via auto-lysosomal pathway (Fig. 5E, F, S5A, B). Furthermore, Western blot analysis revealed that the cellular SQSTM1/p62 and the autophagy membrane LC3b-II/I ratio were increased upon siRNA-mediated Ubr1 depletion in MLC1-S280L cells, as well as in control cells (Fig. 6A, B, S5C). This suggests that Ubr1 has a substantial role in general proteostasis QC. Consistently, the lack of Ubr1 resulted in the accumulation of pre-autophagic isolation membranes with compromised maturation to autophagosomes pinpointing its importance in the initial steps for selective autophagy of MLC1 mutant (Figs. 5C–F, 6E). Supporting the role of SQSTM1/p62 in the recognition of misfolded endosomal MLC1-S280L, the PM-endosomal isolated MLC1-S280L/GlialCAM by cs-IP (as in Fig. 3A) associated with SQSTM1/p62 without Ubr1 and in BafA1 treated cells (Fig. 6C). This interaction of SQSTM1/p62 (and LC3b) to endosomal MLC1-S280L suggested an alternative binding mechanism that is independent of Ubr1.

While Ubr1 is non-essential for the initial recognition of MLC1-S280L by SQSTM1/p62, Ubr1 was required for the maturation of autophagosomes (Fig. 5C–F). The oligomerization of multivalent SQSTM1/p62 allows simultaneous selection of ubiquitinated cargo and pre-autophagic isolation membranes during selective autophagy [64]. Accordingly, the BafA1-induced inhibition of the misfolded endosomal MLC1-S280L transfer to lysosomes led to the disulphide-dependent SQSTM1/p62 oligomerization [65], indicated by their elimination under reducing conditions (Fig. 6D). Notably, the lack of Ubr1 also abrogated the oligomerization of SQSTM1/p62 required for efficient Ub-substrate recognition/oligomerization (Fig. 6D). Collectively, the data so far (Figs. 1, 2, 3, 4, 5 , 6D) support the notion that misfolded MLC1 imposes intracellular Ca^2+^ homeostasis perturbation and renders proteostasis stress to the endosomal pathway, culminating in the activation of Ubr1- and SQSTM1/p62-dependent cargo-selective endophagy.

### Ubiquitination and arginylation cooperatively safeguard endo-lysosomal proteostasis

SQSTM1/p62 was able to bind misfolded endosomal MLC1 without Ubr1 (Figs. 5E, 6D), implying a ubiquitin-independent misfolded cargo recognition mechanism may work in tandem or synergy with the ubiquitin-dependent cargo sorting. SQSTM1/p62 was recently found to bind arginylated (R) proteins that facilitate disulphide bond-linked oligomerization of SQSTM1/p62 and SQSTM1/p62 interaction with LC3, as well as its cargo delivery to the autophagosome [21]. Arginylation can occur on exposed N-terminal residues or internally located side-chains of proteins [66]. To assess this, arginylation was inhibited by tannic acid or depleting arginyltransferase 1 (Ate1) in mutant MLC1 cells and analyzed using immunofluorescence microscopy. Tannic acid (TA) or siAte1 induced the SQSTM1/p62 dispersion prohibiting autophagosome formation in MLC1-S280L cells similar to the lack of Ubr1 (Fig. 6F, G, S5D, E). Inhibition/lack of arginylation and Ubr1 prevented the formation of autophagosomes and enhanced MLC1-S280L and Lamp1 accumulation at the PM (Fig. 6F, G, S5D, E).

**Fig. 7:**
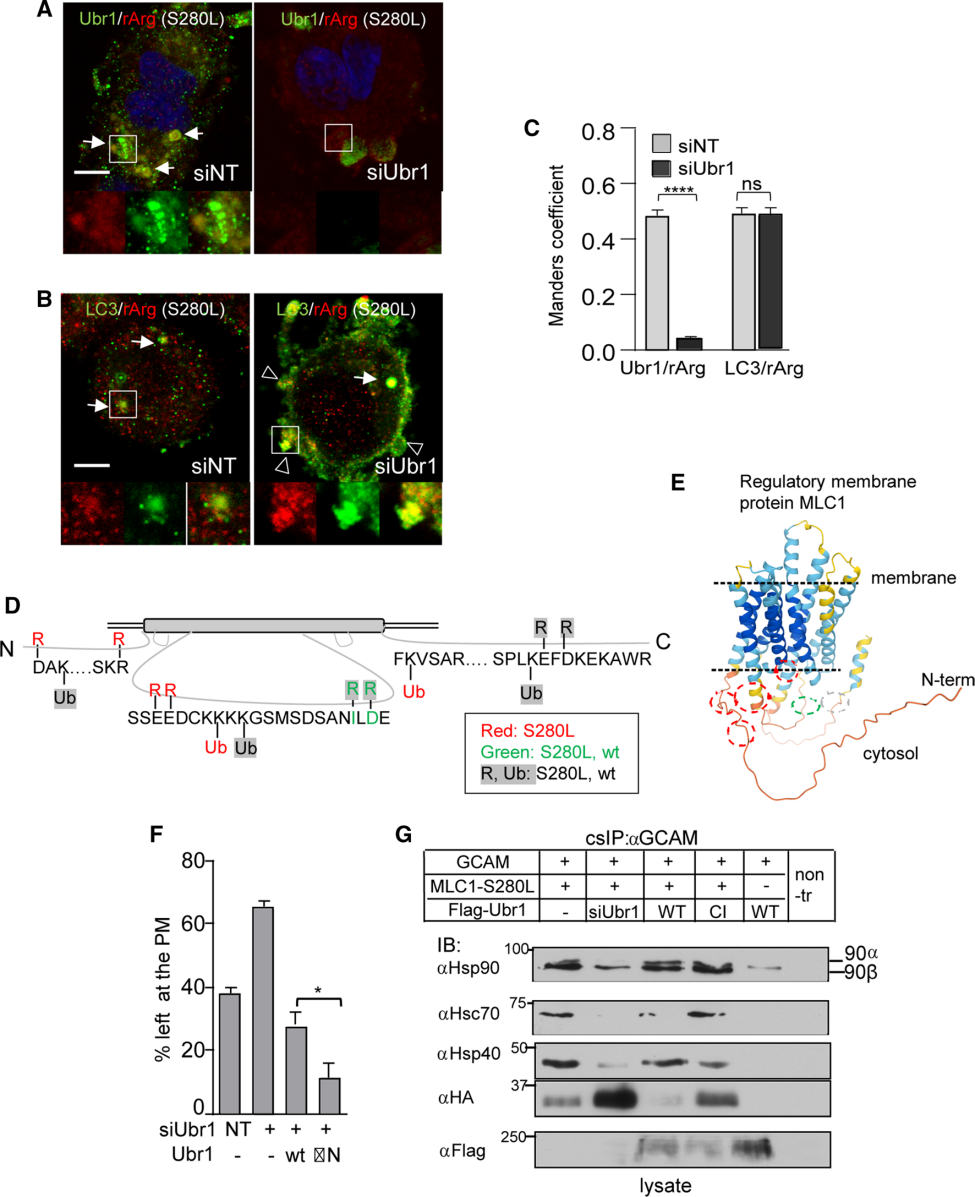
Proximity clustering of ubiquitin/arginine and Ubr1-Hsp90 chaperone complex mediate cargo-selective endophagy. **A** Immunofluorescence microscopy of Ubr1 and arginylated substrate (rArg) colocalization to membrane puncta and autolysosome-like structures in MLC1-S280L expressing U251N cells exposed to siUbr1 or siNT. The insert illustrates the individual staining pattern of the Ubr1/rArg containing structure. Bar 10 µm. **B** Immunofluorescence microscopy of autophagosome marker LC3b and arginylated substrate (rArg) colocalization in MLC1-S280L cells as in **A**. The insert illustrates the individual staining pattern of LC3b/rArg. Bar 10 µm. **C** Manders' overlap coefficient of Ubr1 and arginylated substrates (rArg) (representative **A**) and autophagy marker LC3b with arginylated substrates (rArg) (representative **B**) in MLC1-S280L U251N cells. *n* > 47 cells, ****p < 0.0001, *ns* non-significant. **D** Mass spectrometry analysis of affinity-purified MLC1-wt and S280L amino acids at the intracellular part for arginylation and ubiquitination. Identified peptides are listed in Fig. S5C. **E** Ubiquitin/arginine residues from **C** indicated structural proximity clustering of ubiquitin/arginine residues in misfolded MLC1 (red circles). Red circles: R/Ub S280L, green: R wt/S280L and gray: R/Ub wt/S280L as in **C**. **F** The UBR-box deleted Flag-Ubr1-ΔN and Flag-Ubr1-wt effect on the PM stability of MLC1-S280L was measured using cs-ELISA after 1 h chase. HeLa cells were depleted for Ubr1 or NT using siRNA and Flag-Ubr1 was transiently expressed. **G** The effect of Ubr1 depletion and Flag-Ubr1-wt or -CI expression on Hsp90β/Hsc70/Hsp40-complex association with misfolded MLC1-S280L at the PM-endosomes. Cells were prepared as in E and the PM-endosomal MLC1 was isolated using cs-IP and Western blot analysis as in Fig. 3A. *α* anti, *GCAM* GlialCAM. Means ± SEM, *p* values: *p < 0.05, **p < 0.01, ****p < 0.0001

Arginylation specific antibody toward glutamic (E) or aspartic acid (D) colocalized with Ubr1 to membrane puncta and autolysosome-like structures in S280L cells (Fig. 7A, C). In Ubr1-depleted S280L cells, arginylated substrates preferentially accumulated at the PM with LC3b (Fig. 7B, C) reinforcing the importance of Ubr1 and its activity in selective autophagy.

Both arginylation and Ubr1-induced ubiquitination of misfolded MLC1 were required for the SQSTM1/p62-driven selective endo-phagosome formation. Next, we monitored the arginylation and ubiquitination of the affinity-purified MLC1-wt and S280L by tandem mass spectrometry (Fig. 7C, peptides S5F). Arginylation in S280L but not in MLC1-wt was found D31 and R42 residues in the N-terminal tail and E168-169 residues in the 2nd cytosolic loop of S280L, adjacent to the Ub-acceptor sites (K33, K173 and 175) forming structural proximity clusters (Fig. 7D, red circles). We also observed arginylation in the wt and S280L (Fig. 7D, E, green circle). Ubiquitin in K173 and 175 in the 2nd cytosolic loop and with the C-terminal K360 (Fig. 1A) was also identified with MS/MS, confirming MLC1 ubiquitination results in Fig. 1C, D.

Considering that the recruitment of Ubr1 by arginylation to misfolded MLC1 was limited (Fig. 6E, F, S5D, E), we tested the effect of the N-terminal UBR-box deletion (ΔN) in Ubr1, responsible for the recognition of N-terminal arginylation. ΔN-Ubr1 overexpression accelerated the PM turnover of S280L more than Ubr1-wt, suggesting that Ubr1 is recruited via an alternative mechanism (Fig. 7F). This scenario is consistent with the Hsp70-dependent and N-degron-independent PQC function of Ubr1 in the cytosol [25].

To evaluate the involvement of molecular chaperones in MLC1-S280L recognition by Ubr1, the PM resident or cellular pool of MLC1 was isolated with cs-IP (as in Fig. 3A) or IP, respectively, from siUbr1-depleted and Flag-Ubr1-wt or Ubr1-CI expressing cells followed by blotting for interacting molecular chaperones. Hsp90β (83.2 kDa) preferentially interacted at the PM, while both Hsp90α (90 kDa) and Hsp90β were interacting with the cellular MLC1-S280L (Fig. 7G, S6A). Lack of Ubr1 decreased Hsc70/Hsp40/Hsp90β association with the mutant in both compartments.

In summary, these results show that Ubr1 recruitment to misfolded endosomal MLC1 requires endosomal stress and the Hsp90-chaperone complex. Ubr1-client ubiquitination/arginylation drives SQSTM1/p62 oligomerization promoting cargo-selective endophagy maturation.

### Ubr1/ SQSTM1/p62 clear ubiquitin/arginine PQC clients during Ca^2+^-induced stress

**Fig. 8:**
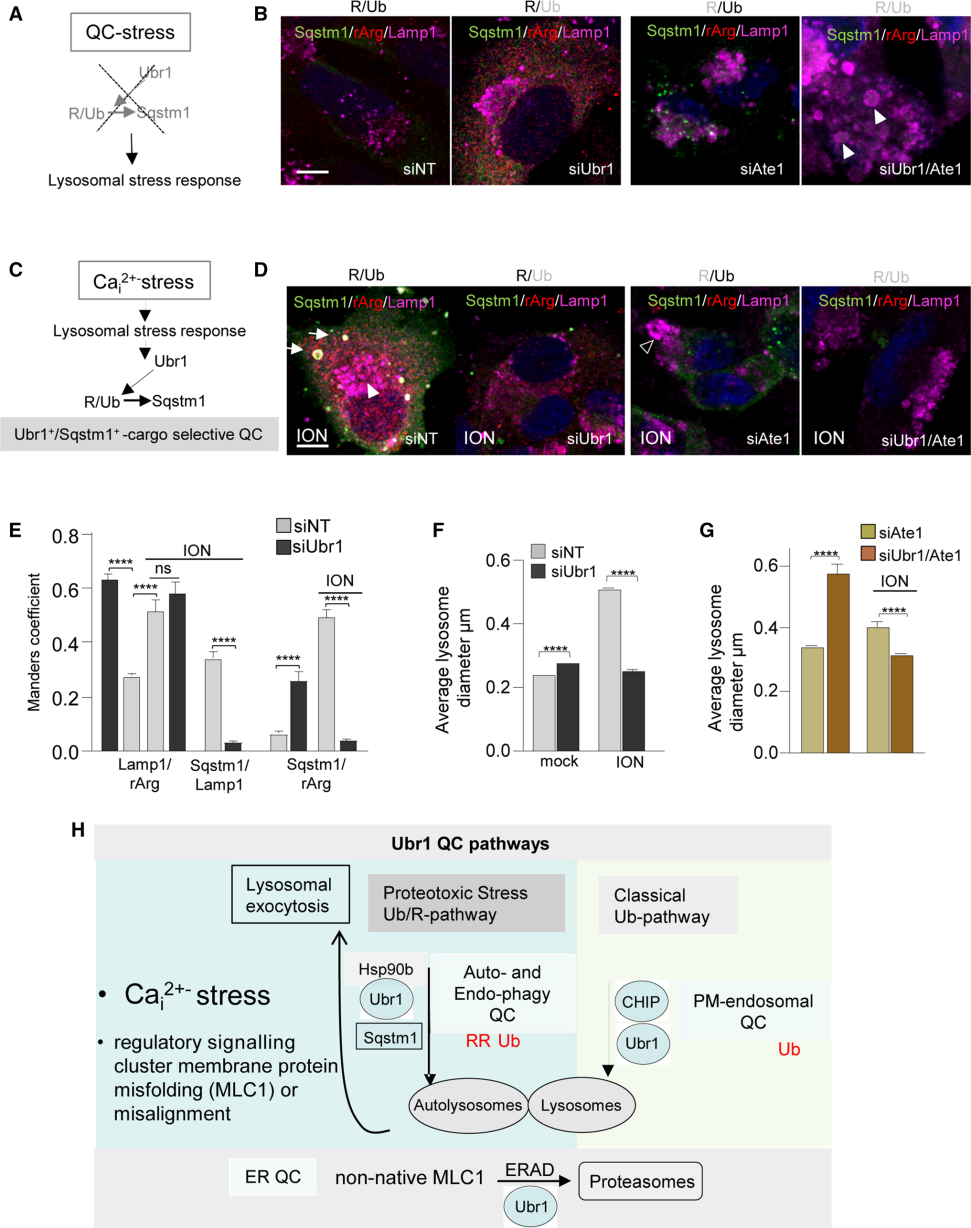
The interplay of ubiquitination and arginylation enhance Ubr1 and SQSTM1/p62-mediated stress QC to safeguard endo-lysosomal pathway proteostasis. **A** Diagram of the proposed role of lack of Ubr1 and arginylation signal resulting in QC-stress. **B** Ubr1 and arginylation protect against endo-lysosomal stress. The morphological analysis of lysosomal response to Ubr1 depletion and/or lack of arginylation by Ate1 with siRNA. SQSTM1/p62 recruitment to lysosomes was determined using immunofluorescence microscopy in Hela cells. Lamp1 was a lysosomal marker. The arrowhead indicates enlarged lysosomes. Top gray labels indicate the inactivation of the arginylation (R) and/or Ubr1 (Ub). Bar 5 µm. **C** Role of Ubr1 and SQSTM1/p62 activation by Ca^2+^-stress signaling in cargo-selective QC. **D** Monitoring Ubr1-dependent Ca^2+^-stress QC as in panel B, but ionomycin (ION) was used to induce intracellular Ca^2+^-stress. The arrow indicates autolysosomes and arrowhead enlarged lysosomes. Bar 5 µm. **E** Manders' overlap coefficient of autophagy scaffold SQSTM1/p62, arginylated substrates (rArg) and lysosomes (Lamp1) in siNT or siUbr1 treated HeLa cells and upon ionomycin Ca^2+^-stress (ION). Representative cells are in panels **B**, **D**. *n* > 48 cells. **F, G** Average lysosomal diameter from experimental conditions presented in **B**, **D**. *n* > 2000 lysosomes. Means ± SEM, *p* values: ****p < 0.0001. **H** Summary of proposed classical ubiquitin (Ub)-mediated QC and ubiquitin/arginine (Ub/R)-mediated Stress and Endo-phagy QC pathways. Ubr1 was found to have a previously unrecognized role as a new Endosomal and Stress QC-ligase at the endosomal autophagy (endophagy) pathway to support PQC during proteostasis stress and disease. Classical ubiquitin (Ub)-mediated QC pathway function toward lysosomes, which uses QC-ligase CHIP, ubiquitin-mediated endosomal cargo concentrating ESCRT machinery and endosomal Ubr1. However, proteostasis stress initiated either by intracellular Ca^2+^ overload or misalignment of the astrocyte regulatory MLC1 signaling cluster cause fused endosomal compartments with altered cargo sorting. This leads to the activation of an alternative Ub/R (arginine)-mediated auto/endophagy QC pathway, where Ubr1 drives ubiquitinated/arginylated clients to autophagy scaffold SQSTM1/P62 to enhance the maturation of cargo-selective autophagosomes. Subsequently, Ubr1 and SQSTM1/p62 function as a rerouting surveillance mechanism to safeguard proteostasis at the endosomal compartments

Finally, we asked whether Ubr1 can attenuate the endosomal stress imposed by the constitutive cellular protein turnover or in association with elevated cytosolic [Ca^2+^] as part of the organellar QC mechanism. Abrogation of Ubr1 activity (Fig. 8A condition) was sufficient to enhance the lysosomal accumulation of arginylated proteins (Fig. 8A, B, E) and lysosomal enlargement (Fig. 8B, F) in the absence of MLC1 expression. Importantly, Ubr1 depletion with inhibition of arginylation by TA or siAte1 provoked pronounced enlargement of lysosomes, indicating that Ubr1 with arginylation is important for endo-lysosomal pathway health (Fig. 8B, F, S6F, H).

Ubr1 is important for limiting the endophagy stress QC caused by MLC1 mutants, coinciding with elevated cytosolic [Ca^2+^] levels. Therefore, we tested whether Ubr1 can clear endogenous clients via cargo-selective SQSTM1/p62 autophagy upon cytosolic Ca^2+^-stress (Fig. 8C condition). Intracellular Ca^2+^ level was elevated by Ca^2+^ ionophore, ionomycin, known to trigger proteostasis stress via mitochondrial oxidative stress and Ca^2+^ egress [67], the release of lysosomal enzymes (Fig. 2D), as well as by reducing PtdIns*(*4,5)P2 concentration critical for the PM protein stability and endocytosis [68], and imposing ER-stress [69]. Ionomycin-induced (ION) the enlargement of Lamp1 + lysosomes, the appearance of Lamp1/SQSTM1/p62 positive autolysosomes and arginylation signal to lysosomes (Lamp1/rArg) with SQSTM1/p62 (Fig. 8D, E). Lack of Ubr1 during Ca^2+^-stress abrogated SQSTM1/p62 expression, autolysosomes and decreased significantly lysosomal diameter (Fig. 8D, F). Inhibition of arginylation by TA or Ate1 depletion alone abrogated selective autolysosome formation and decreased the lysosomal enlargement cooperatively with lack of Ubr1 during Ca^2+^-stress (Fig. 8D, F, G, S6G, H).

In summary, the PQC function of Ubr1 can target ubiquitinated/arginylated clients in SQSTM1/p62-dependent manner for selective endophagy and autophagy during Ca^2+^-stress (summary Fig. 8H and S6I).

## Discussion

Besides the partly non-selective cellular degradation mechanism of autophagy (macro-autophagy), a survival mechanism of eukaryotic cells in nutrient starvation and various stressors, compelling evidence indicates that “selective autophagy” can recognize, sequester and eliminate specifically stressed protein targets.

Here, we describe the previously unrecognized activity of Ubr1 and SQSTM1/p62 in cargo-selective endophagy/autophagy that can clear ubiquitinated and arginylated MLC1 and other endogenous clients under proteostasis stress. We found Ubr1 important for rerouting cargo and alleviating proteostasis stress caused by fused endosomal compartments with altered cargo sorting. The compounded endosomal stress was initiated either by expression of conformationally challenged astrocyte MLC1 signaling cluster variants harboring proximity of ubiquitin-arginine post-translational modifications and/or caused by the cytoplasmic Ca^2+^-overload eliciting proteostasis stress toward endogenous cargo sorting. As a corollary, Ubr1 loss-of-function alone, and especially jointly with arginylation defect, resulted in endo-lysosomal compartments stress.

Although selective autophagy pathways have been recently identified for phase-separated endocytic protein deposits and ubiquitinated internalized MHC-I, and a less selective form to repair or remove acute membrane ruptured endo-lysosomes, they represent mechanistically distinct entities from the Ubr1 and SQSTM1/p62-dependent selective endophagy. Ede1, a yeast homologue of Eps15, is the intrinsic autophagy receptor for the aberrant clathrin-mediated endocytosis assemblies [70], while NBR1 serves as a ubiquitin-binding receptor for the internalized ubiquitinated MHC-I via LC3 to capture nascent autophagic vesicles [71]. Galectin-dependent recruitment of ESCRTs to endo-lysosomes to transiently recover acute membrane damage or subsequent galectin-initiated autophagy represents a non-selective response to endo-lysosomal membrane tiers [72–74]. Here, we demonstrated that the Ubr1-chaperone complex can recognize ubiquitinated and arginylated misfolded endosomal MLC1 variants by the SQSTM1/p62 (and LC3) autophagy receptor to mediate endophagy. This selective stress autophagy mechanism is universal as a variety of arginylated Ubr1-client proteins were required to preserve the endosomal compartments health during Ca^2+^-overload. Whether Ubr1 targets are activated by Ca^2+^- and/or other stressors require further investigation.

Dual post-translational modifications in the QC by organelle autophagy are not without precedent and likely serve to enhance the fidelity of structurally impaired protein elimination. Amplified mitophagy relies on both phosphorylation and ubiquitination by PINK1 and QC E3-ligase Parkin in Parkinson’s disease [16]. The alpha-1 antitrypsin variant was sequestered as a ubiquitinated and arginylated complex by SQSTM1/p62 for ER-phagy [17, 18]. Selective endophagy requires sequential ubiquitin-arginine modifications and is likely initiated at Rab11A + early endosomes with the association of PI3P [28] by a critical platform for the adaptor WIPI2, ATG16L1, ATG9 and LC3 conjugation with dynamin 2-dependent endosomal tubule scission, leading to autophagosome formation [75–77]. Rab11 + membranes were at autophagosome engulfment sites for cytosolic mutant huntingtin exon 1 and damaged mitochondria (mitophagy) in complex with SQSTM1/p62 [28]. Rab11 influences cargo recycling between early and recycling endosomes, and rerouting damaged cargoes from recycling is prudent for protecting the homeostasis of the PM and late endosomes/lysosomes [61, 78, 79].

Only a few Ub QC ligases have been identified at the PM-endosomes or for proteostasis stress. CHIP relies on the Hsc70/Hsp90-dependent client recognition, while the endosomal RFFL binds conformationally defective substrates independently of molecular chaperones [6, 7, 9, 11]. The yeast Rsp5 [14] limits the PM accumulation of heat-stressed proteins in concert with arrestin-related adaptors [80]. Ubr1 has been invoked in chaperone-dependent and -independent [25, 26, 81], and both stress-induced cytosolic and ER QC in yeast [24]. Unlike CHIP, Ubr1 has not been associated with Stress QC and membrane protein QC at the PM-endosomes in higher eukaryotes. Our results also demonstrate that Ubr1 is a QC-ligase that has a role in the ERAD of conformationally defective MLC1s similar to a previously found role for CFTR expressed heterologously in yeast [27] and endophagy QC during Ca^2+^ proteostasis stress in human cells.

The dynamics of molecular chaperones and proteostasis stress may play a role for both CHIP and Ubr1 in QC. CHIP loss-of-function increases sensitivity to senescence, stress and aging [82–84]. Our findings link Ubr1 stress QC to a major biological disease pathway of Ca^2+^ signaling, endo-lysosomal membrane stress and selective autophagy with implications to a variety of human diseases [85, 86]. Thus, stress-activated Ubr1 can constitute an important backup QC mechanism toward certain clients. Our data and a recent report [24] suggest alternative mechanisms for the stress QC outside Hsc70-CHIP complex recognition or Ubr1 N-recognin property toward destabilizing amino acids [87]. Upon acute stress, CHIP can sense Hsp70 deficiency and relocate to support membrane organelles QC independently of chaperones [88]. Osmotic stress activates Ubr1 independently of the UBR-box to degrade some misfolded cytosolic and ER membrane proteins in yeast [24] consistent with its N-recognin-independent function [25, 81]. We found that the lack of UBR-box in Ubr1 enhances QC, Ubr1 is recruited with Hsp90-complex and is a requirement for SQSTM1/p62 oligomerization, underscoring the role of Ubr1 stress QC in human cells. Diversification of Ubr1 recognition capacity, arginylation and recruitment of SQSTM1/p62 during proteostasis stress may expand the limited substrate specificity and capacity of the mammalian QC ligases.

The plethora of genetic mutations, including in risk genes for cancers and neurodegenerative diseases, underscores the importance of surveillance mechanisms to preserve proper protein/membrane flow along the endo-lysosomal pathway. The identified Ubr1 QC network advances our understanding of alleviating stressed protein/membrane flow and Ca^2+^-stress clients toward selective auto/endophagy. Our results highlight the role of ubiquitination and arginylation in the stress QC and offer novel therapeutic targets in diseases afflicting membrane proteins and organelle proteostasis.

## Materials and methods

### Experimental models

Parental or inducible Lenti-X Tet-On [6] HeLa and U251N cells with and without 2HA-MLC1 (GeneID: 23209) expression were used in experiments and cultured under standard conditions [13, 37, 38]. Lentivirus production, transduction and doxycycline induction [6] were done as described before [37] as well as transfection of GlialCAM-Flag [13, 37]. Transfection of plasmid DNA was performed using Lipofectamine 2000 (Thermo Fisher Scientific) and siRNA using RNAiMAX or Oligofectamine transfection reagent (Thermo Fisher Scientific). Thermolabile E1 mutant CHO cells *ts20* and control E36 cells were preincubated at 40 °C for 3 h to inactivate E1 [7]. Flag-Ubr1-wt in pCMV-Tag2B was a gift from Y.Yamaguchi (GeneID: 499877). C1011 was mutated to alanine to generate inactive Ubr1 ligase (Ubr1-CI). To create a mCherry vector, Ubr1-wt and Ubr1-CI were transferred to pmCherry-C1 (Clonetech).

### Immunoprecipitation and protein analyses

In total cell IP, the Ab was added directly to cell lysates. Selective isolation of MLC1-complex from the PM was achieved by cs-IP using anti-GlialCAM Ab (1:2000, R&D Systems). Ab was bound to live cells for 45 min on ice, after which the unbound Ab was washed off. Cells were lysed in Triton X-100 lysis buffer (1% Triton X-100, 25 mM Tris–Cl, 150 mM NaCl, pH 8.0, 10 μM MG132 containing 20 μM PR-619, 10 μg/ml pepstatin + leupeptin, 1 mM phenylmethylsulfonyl fluoride, and 5 mM *N*-ethylmaleimide) on ice or in co-IP assays, a milder lysis buffer was used by changing Triton X-100 to 0.4% NP-40. For detecting direct ubiquitination of MLC1, lysates were denatured using 1% SDS for 5 min, after which the SDS concentration was adjusted to 0.1%. The second IP step was performed using anti-HA (Biolegend, for MLC1). Treatment with cycloheximide (100 μg/ml) was carried out in full medium at 37 °C for indicated times. BFA treatment (5 μg/ml) was done in the full medium at 37 °C for 20 h. Autophagosome-lysosome fusion was inhibited by adding Bafilomycin A1 (200 nM) and proteasomes with Bortezomib (1 μM) for indicated times. Molecular chaperone Abs were anti-Hsp90, Hsc70 and Hsp40 (DNAJB1) (Enzo Lifesciences), ESCRT Abs anti-TSG101 (Santa Cruz), -Stam (Santa Cruz), -Hrs (Santa Cruz), other Abs anti-Na^+^K^+^ATPase (Abcam), -Ubr1 (Abcam), -SQSTM1 (Abcam), -LC3b (Genetex, Merck) and P4D1 anti-Ub Ab (Santa Cruz).

Cross-linking of the PM-endosomal MLC1 to Ubr1 was performed using dithiobis(succinimidyl propionate) in 0.05 mM (Thermo Fisher Scientific) for 10 min at room temperature and cs-IPed as above. Proximity biotinylation based on method [89] between MLC1 and Ubr1 was performed 24 h with 50 µM biotin and cs-IP was performed as above. Eluate was diluted to Triton X-100 lysis buffer containing 0.4% SDS. Biotinylated proteins were isolated using a Streptavidin (Thermo-Fisher Scientific) pull-down assay. Beads were collected and washed with lysis buffer four times and twice with 1 ml of 2% SDS in dH_2_O. Bound proteins were removed from the magnetic beads with 50 µl of Laemmli SDS-sample buffer saturated with 20 mM biotin at 98 °C. For all assays, proteins were separated using SDS-PAGE and Western blot analysis. Densitometric analyses were done measuring signal intensity at the linear range using Image Studio Lite (LiCOR) or Fiji (National Institutes of Health). When quantification was done for the IP samples, the signal was normalized for the amount of precipitated MLC1.

### Post-translational modification identification by mass spectrometry

Affinity purification of MLC1 was done using anti-HA in a lysis buffer as described above and final washing twice in 50 mM NH_4_HCO_3_. Beads were resuspended to 20 mM Tris–HCl (pH 8.0) and 750 ng of trypsin was added for 24 h, and an additional 250 ng for 3 h, and incubated at 37 °C. Peptides were lyophilized and formic acid was added in 2%. LC–MS/MS was done on the Orbitrap Fusion Tribrid Instrument connected to an UltiMate 3000 UHPLC liquid chromatography system (Thermo-Fisher Scientific). The acquired raw files of mass spectra were searched against the Uniprot human database and analyzed using Byonic or MaxQuant with dynamic modifications for ubiquitination (+ 114.04293 Da) and arginylation (+ 156.10111). The false discovery rate (FDR) was set to 1%.

### Live-cell ELISA

The PM/endosomal protein expression and turnover were measured using the PM epitope labeling and cs-ELISA in live cells [6, 7, 38]. Endosomal internalization was measured for 5 min and turnover for indicated times at 37 °C. TfR was measured using horseradish peroxidase (HRP)-conjugated transferrin (Thermo Fisher Scientific) and CD4 with anti-CD4 (BD Pharmigen). The transferrin-HRP or HRP-conjugated secondary Abs were measured either by luminescence using HRP-Substrate (SuperSignal West Pico, Thermo Fisher Scientific) or Ampilite (ATT Bioquest) or Amplex Red assay (Thermo Fisher Scientific).

Differences in cytosolic Ca^2+^-fold levels were measured using Calbryte 520 AM (5 μM) (ATT Bioquest). The dye was loaded into cells for 45 min at 37 °C and 30 min at RT after which 1 mM Probenecid was added to each well before the fluorescence plate reading.

### Lysosomes exocytosis and enzyme assays

The lysosomal exocytosis of Lamp1 to the PM or lysosomal beta-hexosaminidase secretion was measured in MLC1-wt or misfolded variant expressing cells. Parental and potassium channel hERG expressing cells [6] were used as controls. The PM appearance of the Lysosomal marker Lamp1, indicating lysosomal exocytosis, was measured by live-cell cs-ELISA, using extracellular anti-Lamp1 Ab (Abcam). Beta- hexosaminidase secretion was monitored using a fluorometric assay. Medium or cell lysates were diluted in 0.1 M sodium citrate buffer, pH 4.5, and incubated with the 0.1 mM substrate methylumbelliferyl-2-acetamido-2-deoxy-b-D-glucopyranoside (Sigma) for 1 h at 37 °C. The reaction was stopped by the addition of 0.5 M sodium glycine buffer, pH 10.5. Hexosaminidase activity was measured using the release of fluorescent 4-methylumbelliferone at 360 nm excitation wavelength and the 460 nm emission wavelength. The secreted enzyme amount was calculated relative to the total cellular enzyme normalized for cellular proteins. Ionomycin induction was used to increase calcium-dependent lysosomal exocytosis/secretion 5 μM for 1 h at 37 °C before measurements.

### Microscope imaging

The pH of endocytic vesicles containing indicated cargo molecules was measured using live-cell single vesicle fluorescence microscopy and image analysis as described previously [6, 7, 13, 37, 59, 60]. Membrane protein cargo was labeled sequentially with appropriate primary Ab against extracellular epitope and with FITC-goat anti-mouse secondary Fab (Jackson Immunoresearch). Internalization was initiated at 37 °C and allowed to continue for indicated times. Recycling endosomes were labeled with 5 µg/ml FITC-Tf (Jackson Immunoresearch) for 1 h. At least > 250 vesicles from 25 to 50 cells per experiment were analyzed, and the average weighted mean was calculated for at least three or more independent experiments. The analysis was performed on an inverted fluorescence microscope Nikon TI-E equipped with Lumencor Spectra X light source and electron-multiplying charge-coupled device (Photometrics) equipped with an Evolve 512 electron-multiplying charge-coupled device (EM CCD) camera (Photometrics Technology) and a 63 × /1.4 numerical aperture (NA) Plan Apochromat oil-immersion objective. The acquisition was performed at 490 ± 5 and 440 ± 10 nm excitation wavelengths, using a 535 ± 25 nm emission filter and was analyzed with NIS-Elements (Nikon).

For confocal colocalization microscopy (Leica TCS SP8X confocal microscope or LSM780 microscope, Carl Zeiss MicroImaging, 63 × /1.4 NA Plan Apochromat oil-immersion objective) cells were cultured in 100 µg/ml poly-l-lysine coated coverslips. For the PM staining or endo-lysosomal cargo visualization, the PM proteins were labeled in live cells and allowed to internalize for indicated times. Cells were fixed with 4% paraformaldehyde in PBS for 15 min. Intracellular antigens were visualized in fixed, permeabilized cells using the indicated primary Abs in PBS-0.5% BSA for 1 h at room temperature. Following antibodies were used: anti-EEA1 (Cell Signaling), -Flag (M2, Sigma), -ERp57 (GeneTex), -LAMP1 (Abcam, Novus Biologicals), -LAMP2 (Abcam), -CLC (Sigma), -AP2 (Sigma), Ubr1 (Abcam), -SQSTM1 (Abcam), -LC3b (Genetex, Merck), -R-Bip (Merck), -Arg (Thermo Fisher Scientific), Phalloidin-Alexa 594 (Thermo Fisher Scientific). Secondary antibodies were from Thermo Fisher Scientific (Anti-Mouse Alexa 488, Anti-Rabbit Alexa 555, Anti-Rabbit Alexa 488, Anti-Goat Alexa 647) or Jackson Immunoresearch (Anti-Rat Alexa 647, Anti-Rat Dylight 405).

Proximity ligation assay was performed using Duolink-technology following the manufactures instructions. For endosomal detection, anti-HA Ab (Biolegends, for MLC1) was allowed to internalize in live cells. For detecting cell surface MLC1, cells were fixed before Ab binding. After MLC1 internalization or cell surface labeling, intracellular proteins Ubr1 and EEA1 were detected on fixed samples. Anti-mouse plus and anti-rabbit minus probes were used to crosslink the desired two epitopes and visualized as described above. Manders' overlap coefficient was quantified from 25 to 200 cells and Lamp + lysosomal diameter from > 2000 organelles using Fiji-plugins.

Cytosolic Ca^2+^ concentration was measured in cells plated on glass coverslips and loaded with Fura2-AM (5 µM, Thermo Fisher Scientific) for 30 min, washed and incubated in Krebs–Ringer–Hepes solution for 10 min at 37 °C. The signal was measured using a Nikon TE300 Eclipse microscope equipped with a Sutter DG-4/OF wavelength switcher, Omega XF04 filter set for Fura-2, Photonic Science ISIS-3 intensified CCD camera and MetaFluor software. Images were obtained every 20 s using a 20X objective. Ratio values (340/380 nm) were transformed to cytosolic [Ca^2+^] using the equation derived by [90].

### Statistics

Paired or unpaired two-tailed Student’s *t* test was used for p values as indicated in the figure legends. Statistical significance was set to *p* < 0.05. All data in curves and bar plots represent the means average of at least three or more independent experiments. Data are expressed as means ± SEM.

## Supplementary Information

Below is the link to the electronic supplementary material.

Supplementary file1 (PDF 2172 KB)

## Data Availability

The datasets generated during the current study are available from the corresponding author on reasonable request.

## Abbreviations

α: Anti
Ate1: Arginyltransferase 1
CHIP: C-terminus of Hsc70-interacting protein
cs: Cell surface
ER: Endoplasmic reticulum
ERAD: Endoplasmic reticulum-associated degradation
ESCRT: Endosomal sorting complex required for transport
GlialCAM: Glial cell adhesion molecule
IP: Immunoprecipitation
LAMP1: Lysosomal associated membrane protein 1
MLC1: Megalencephalic leukoencephalopathy with subcortical cyst
ns: Non-significant
NT: Non-target
PM: Plasma membrane
PQC: Protein quality control
siRNA: Small interfering RNA
Ub: Ubiquitin
Ubr1: Ubiquitin-protein ligase E3 component N-recognin 1
