# Supplementary material for: Ubr1-induced selective endophagy/autophagy protects against the endosomal and Ca2+-induced proteostasis disease stress
Source: Cell Mol Life Sci. 2022 Mar 1;79(3):167. doi: 10.1007/s00018-022-04191-8 (PMC8888484; doi:10.1007/s00018-022-04191-8)
Supplement: Supplementary file 1 — Supplementary file1 (PDF 2172 KB) [file 18_2022_4191_MOESM1_ESM.pdf]

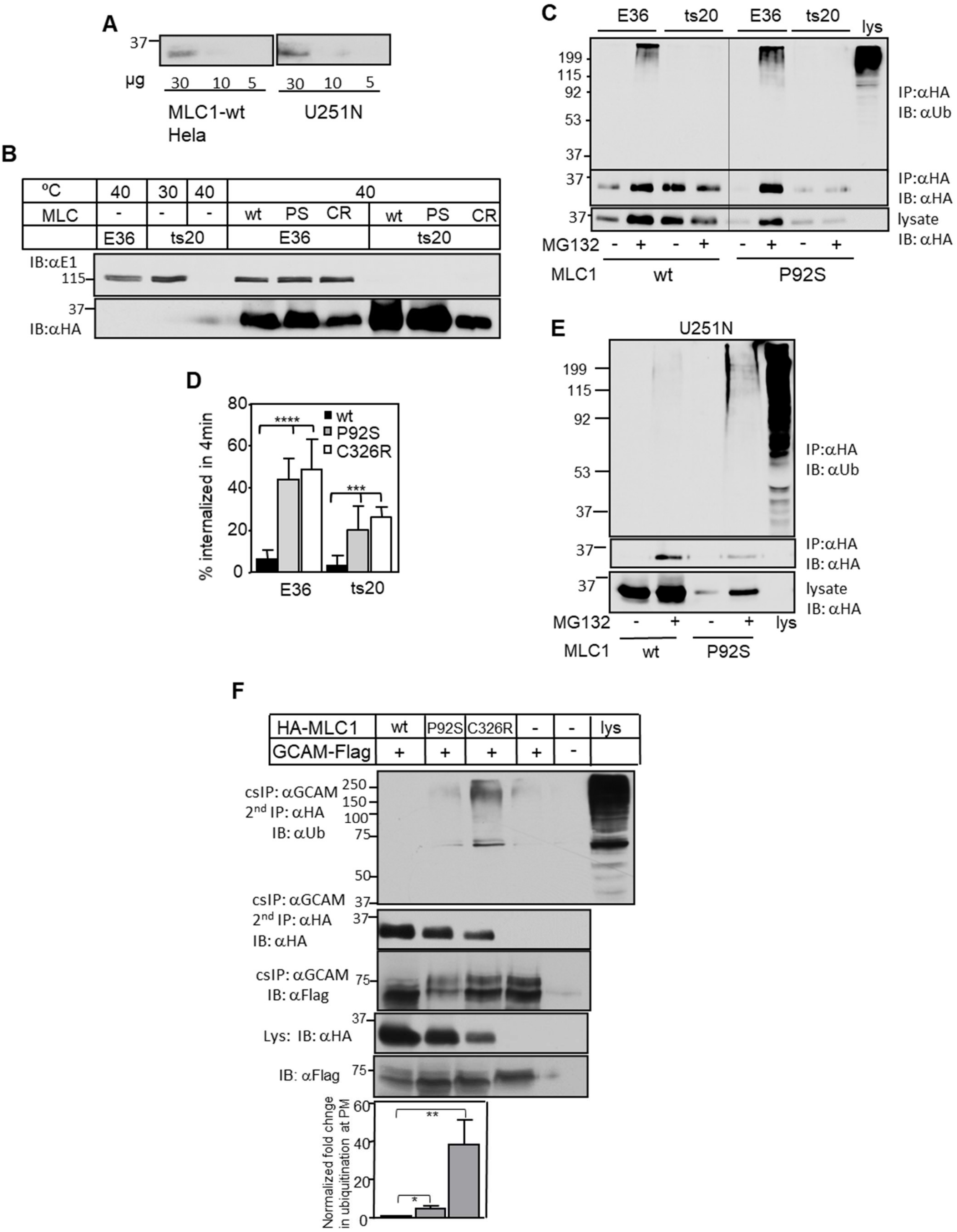
A
37
μg
30 10 5 30 10 5
MLC1-wt Hela
U251N
B
ºC 40 30 40 40
MLC - - - wt PS CR wt PS CR
E36 ts20 E36 ts20
IB:αE1
115
37
IB:αHA
C
E36 ts20 E36 ts20 lys
199
115
92
53
37
IP:αHA
IB: αUb
37
IP:αHA
IB: αHA
37
lysate
IB: αHA
MG132 - + - + - + - +
MLC1 wt P92S
D
% internalized in 4min
80
60
40
20
0
****
***
wt
P92S
C326R
E36 ts20
E
U251N
199
115
92
53
37
IP:αHA
IB: αUb
37
IP:αHA
IB: αHA
37
lysate
IB: αHA
MG132 - + - + lys
MLC1 wt P92S
F
HA-MLC1 wt P92S C326R - - lys
GCAM-Flag + + + + -
csIP: αGCAM
2nd IP: αHA
IB: αUb
250
150
100
75
50
37
csIP: αGCAM
2nd IP: αHA
IB: αHA
37
csIP: αGCAM
IB: αFlag
75
37
Lys: IB: αHA
IB: αFlag
75
Normalized fold chnge in ubiquitination at PM
60
40
20
0
**
*

Figure S1. Astrocyte membrane regulatory cluster MLC1 disease variants are ubiquitinated at the PM-endosomes

A) Immunoblotting of HA-MLC1-wt expression in stable inducible HeLa to endogenous MLC1 in astrocytic U251N cells using an anti-MLC1 antibody. The lysate amount is indicated. **B-D)** The depletion of the temperature-sensitive (ts) E1 ubiquitin-activating enzyme impeded the MLC1 ubiquitination and decreased PM turnover. HA-MLC1-wt and disease associated mutations P92S (PS) and C326R (CR) were transiently expressed in E36 control and ts20 cells and the E1 was inactivated at 40ºC for 3h, and the total protein expression was analyzed by Western blotting (B). Ubiquitin conjugates in MLC1 and inhibition of degradation with MG132 were done as in A (C). Cs-ELISA was performed to detect changes in the endosomal internalized (4min) amount of MLC1 (D). **E)** Misfolded MLC1 is more ubiquitinated than the wt in astrocytic U251N cells. Immunoprecipitation with anti-HA was performed for MLC1-wt and P92S mutant in denaturing conditions to detect direct ubiquitination of MLC1. Degradation of MLC1 was inhibited with MG132 for 2h to increase the detection of ubiquitination. Stable HA-MLC1 astrocytic U251N cells were used. Lys, lysate. **F)** The PM-endosomal MLC1 misfolding increases ubiquitination. The MLC1/GlialCAM complex was first cell surface immunoprecipitated (cs-IP) using GlialCAM Ab and the second IP was performed with HA Ab for MLC1 after denaturation. GlialCAM-Flag was transiently expressed in stable inducible HA-MLC1 HeLa. The ubiquitination was normalized to the MLC1 amount at the PM. GCAM, GlialCAM; lys, lysate; α, anti. Means ± SEM, n≥3. p-value: *<0.05, **<0.01, ***<0.001 ****<0.0001.

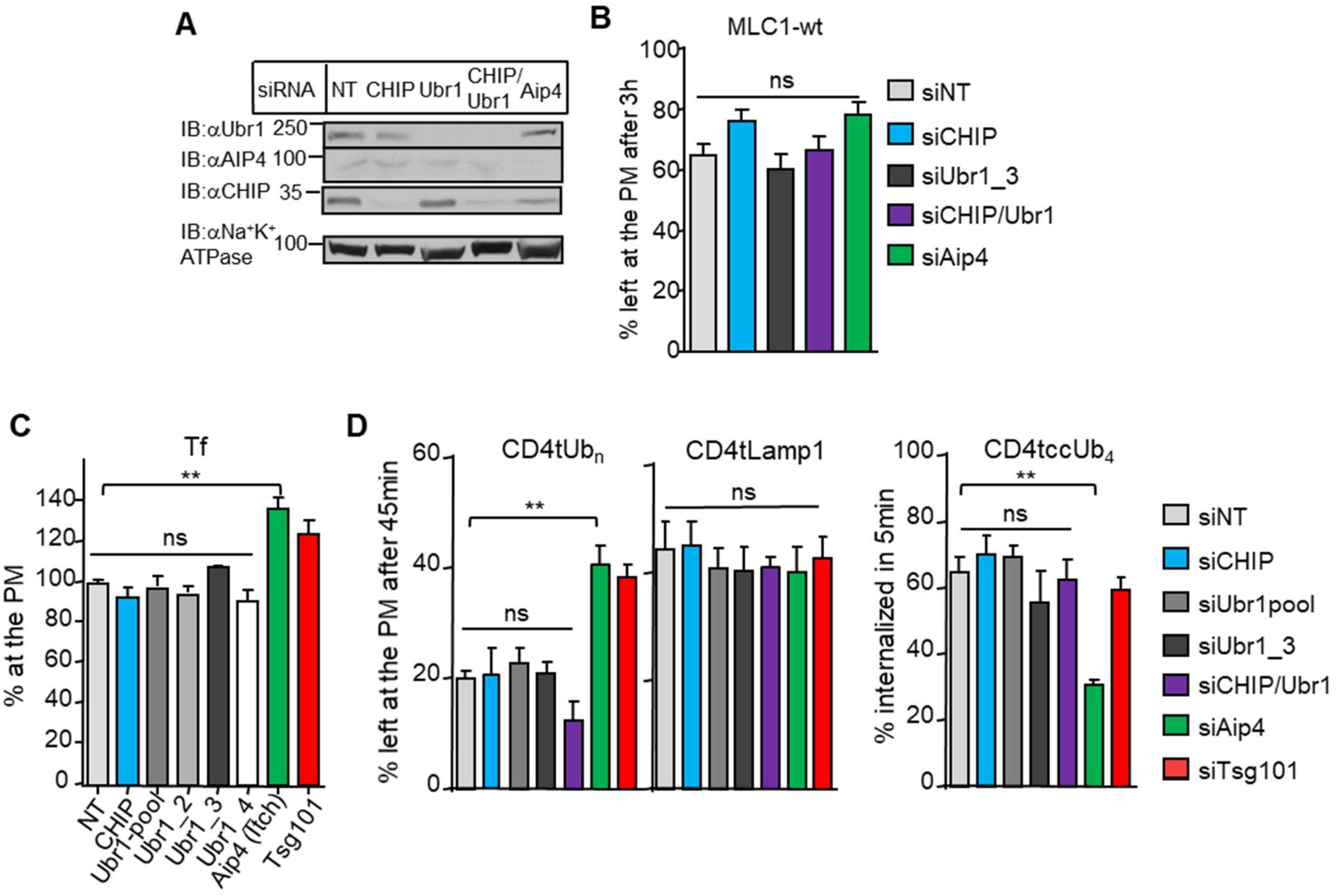

Figure S2. CHIP and Ubr1 are post-Golgi QC E3 ubiquitin ligases

A) Western blot analysis of siRNA cells from Fig.2E depleted for candidate QC E3 ligases. **B)** Candidate E3 ligase depletion effect on native HA-MLC1-wt stability at the PM was measured using cs-ELISA expressed in stable inducible HeLa. Differences are not statistically significant (ns). **C)** Candidate E3 ligase depletion effect on cell recycling endosomes. Internalization was measured for transferrin receptor (Tf) using cs-ELISA. SiNT served as a negative and siTsg101 as a positive control. Only Aip4 (Itch) had a significant effect. **D)** Candidate E3 ligase depletion (siRNA) effect on constitutively polyubiquitinated (CD4tUb$_n$), lysosomal targeted (CD4tLamp1) or tetra-ubiquitinated cargo (CD4tccUb$_4$) in stable HeLa. The PM turnover was measured for CD4tUb$_n$ and CD4tLamp1, and endosomal internalization (5min) for CD4tccUb$_4$ due to its fast turnover. Only Aip4 (Itch) had a significant effect on ubiquitinated cargo (CD4tUb$_n$ and CD4tccUb$_4$) when compared to NT control indicating non-specific ubiquitinated cargo recognition. NT, non-target, Means ± SEM, n≥3. p-value: *<0.05, **<0.01, ***<0.001 ****<0.0001.

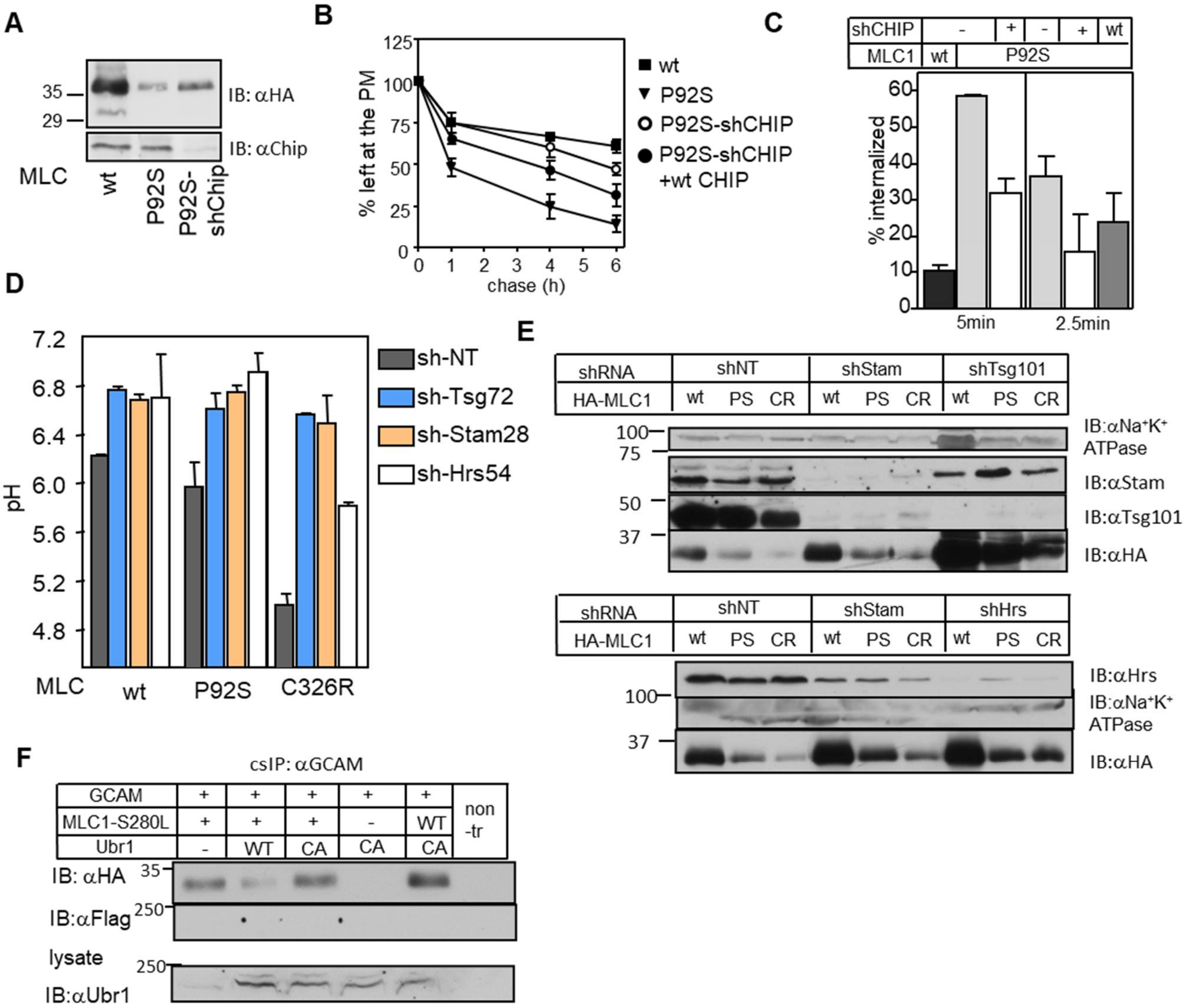

Figure S3. Classical CHIP and ESCRT-mediated ubiquitin-dependent endo-lysosomal QC pathway

A-C) The depletion of molecular chaperone-dependent E3 ubiquitin ligase CHIP decreased the PM turnover of misfolded MLC1. shNT or shCHIP cells were transiently transfected with disease associated with mutant P92S or MLC1-wt. Western blot analysis of total cellular lysates was done to detect total protein expression (A), and cs-ELISA to detect changes in the PM turnover (B) and internalization (C). **D**) The depletion of ESCRT0-I components impaired the lysosomal targeting of misfolded MLC1 mutants. Vesicular pH measurement was performed for MLC-wt or disease associated mutant P92s or C326R containing endosomes. Vesicular pH of MLC1 cargo containing endosomes was determined in cells depleted for Tsg101, Stam2 or Hrs or control shNT. Mean vesicular pH after 1h chase is shown. The values are means ± SEM of 3 independent experiments. **E**) Western blot analysis of shESCRTs and shNT cell lines used in I for MLC1-wt or misfolded mutants P92S or C326R. (**F**) Association of Ubr1 with misfolded MLC1-S280L. Cells expressing S280L mutant were overexpressed with Flag-Ubr1-wt or CA. Cross-linking was done with dithiobis(succinimidyl propionate), after which the cs-immunoprecipitation of the MLC1/GlialCAM -complex was performed. Ubr1 association with S280L was seen faintly in lanes 2-3. MLC1-wt was used as a folded native control.

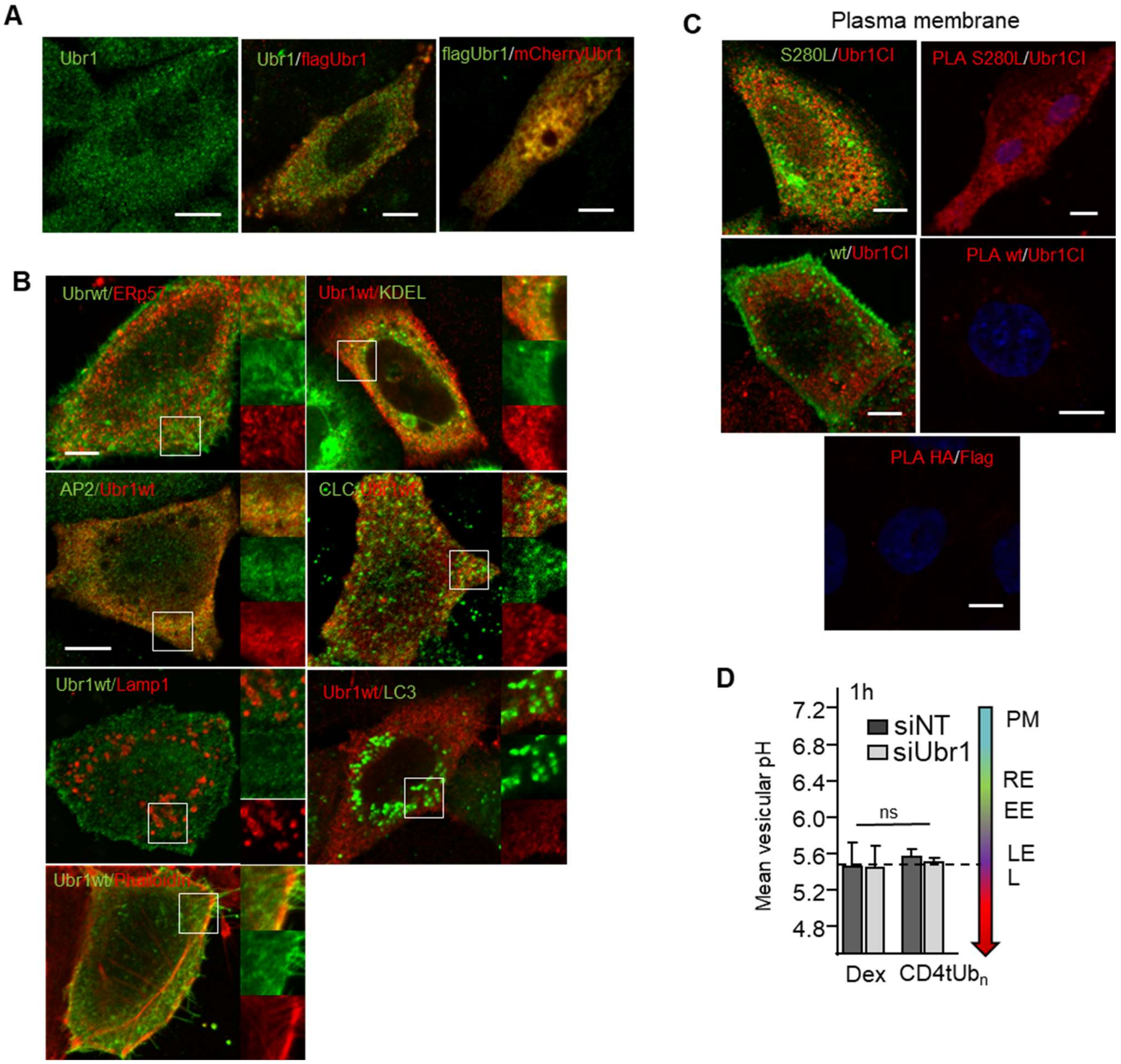

Figure S4. Subcellular location of Ubr1

A) Expression of Ubr1 was detected by using anti-Ubr1 (endogenous) or expressing Flag-Ubr1-wt or mCherry-Ubr1-wt. The appearance of nuclear Ubr1 varied and maybe cell cycle-dependent. Bar 5 µm. **B**) Ubr1 expression in different subcellular markers was monitored using Flag-Ubr1-wt and markers for the ER (anti-ERp57 or GFP-KDEL), F-actin at endosomal invagination sites (phalloidin), endosomal clathrin-coated vesicles (AP2 and CLC), lysosomes (Lamp1) or autophagy (GFP-LC3). Bar 5 µm. **C**) Interaction of MLC1-S280L and Ubr1 at the PM. The S280L at the PM was labelled without internalization with flag-Ubr1-CA, and PLA was performed between HA-Flag epitopes. MLC1-wt and cells without S280L expression were used as a control. Bar 5 µm. **D**) Controls for Ubr1 specificity toward misfolded MLC1 (as in Fig.S2). Fluid phase marker dextran (Dex) and poly-ubiquitinated model (CD4tUb$_n$) were used as control cargoes for siUbr1. Mean vesicular pH measurement of cargo labelled endosomes was determined using live-cell microscopy in cells depleted for Ubr1 or control siNT. PM; plasma membrane, RE; recycling endosome (pH 6.3-6.2), EE; early endosomes, LE; late endosome, L; lysosome.

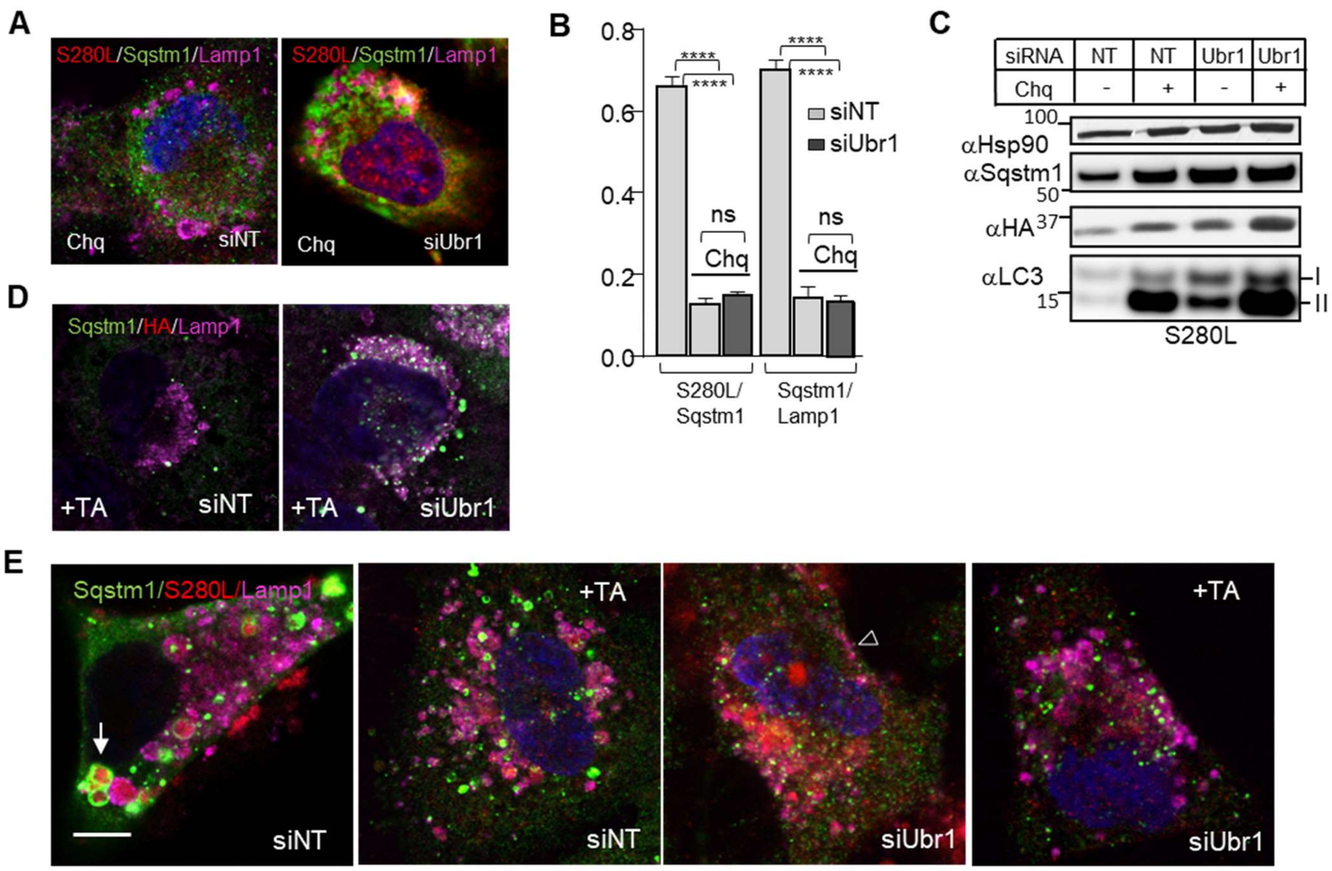

F

MLC1	identified peptide	PTM
N-term	R.GRQDPASYAPD+[+156.1011]AK[+114.04]PSDLQLSK.R	Ub, R
	R.QDPASYAPD[+156.1011]AK[+114.04]PSDLQLSKR[+156.1011].L	Ub, R
1st loop	TV.SRRNANVIPN FQ	none
2nd loop	R.SSE[+156.1011]E[+156.1011]DCKK[+114.043].K	R, R, Ub
	K.K[+114.043]GSMSDSANILDEVPFPAR.V	Ub
	K.GSMSDSANILDEVPFPAR	none
	K.GSMSDSANI[+156.1011]LD[+156.1011]EVPFPAR.V	R, R
3rd loop	AA ECPSKCLVEV	none
C-term	R.FK[+114.043]VSAR.L	Ub
	R.SPLK[+114.043]EFDK.E	Ub
	.E[+156.1011]FD[+156.1011]KEKAWR.A	R, R,

Figure S5. Proteotoxic stress induces Ubr1, ubiquitination, arginylation and autophagy to safeguard endo-lysosomal pathway proteostasis

A) Related to Fig. 5E-F. The effect of Ubr1 and endosomal MLC1-S280L on the endosomal stress and recruitment of autophagy scaffold SQSTM1/p62 to phagophores in MLC1-S280L cells. The endo-lysosomal pathway morphology was monitored by immunofluorescence microscopy in siRNA-treated cells for Ubr1 or control NT. Chloroquine (Chq) was used to inhibit autophagosome-lysosome fusions, which resulted in the cytosolic dispersion of

SQSTM1/p62. Endosomal MLC1-S280L was labelled by anti-HA Ab capture for 30min (37°C) and visualized by anti-mouse secondary Ab. Lamp1 was used as a lysosomal marker to detect the enlarged lysosomal phenotype. Bar 5 µm. **B)** Manders' overlap coefficient of selective autophagy scaffold SQSTM1/p62 and lysosomes (Lamp1) with HA-MLC1-S280L. Representative cells for siNT are in Fig.5E and QC cells in A. n>30 cells. Means ± SEM, p-value: ****p<0.0001, ns; non-significant. **C)** Related to Fig.6A. Western blot analysis of cells depleted with siRNA for Ubr1 or NT to measure the expression of SQSTM1/p62 and the formation of mature autophagosomes using lipidated/cytosolic LC3b-II/I ratio in HA-MLC1-S280L and non-expressing U251N cells. QC was used to inhibit autophagosome-lysosome fusions to decrease autophagosome-cargo degradation. Hsp90 was a loading control. **D-E**) The connection of Ubr1 to arginylation and SQSTM1/p62 recruitment in MLC1-S280L expressing HeLa cells (B) and in control U251N cells (A). When indicated, Ubr1 was depleted with siRNA, tannic acid (TA) was used to inhibit arginylation and Lamp1 to mark lysosomes. The arrow indicates autolysosomes/autophagosomes and arrowheads lysosomal Lamp1 at the PM. Manders' overlap coefficient for A is in main Fig5G. Representative figures are in A-B. n>37 cells. Bar 5 µm. **F**) Mass spectrometry analysis of affinity-purified MLC1-wt and S280L amino acids at the intracellular part for arginylation (R) and ubiquitination (Ub). Intracellular peptide fragments, their location in the MLC1 and modifications are indicated. The schematic figure is in 7C.

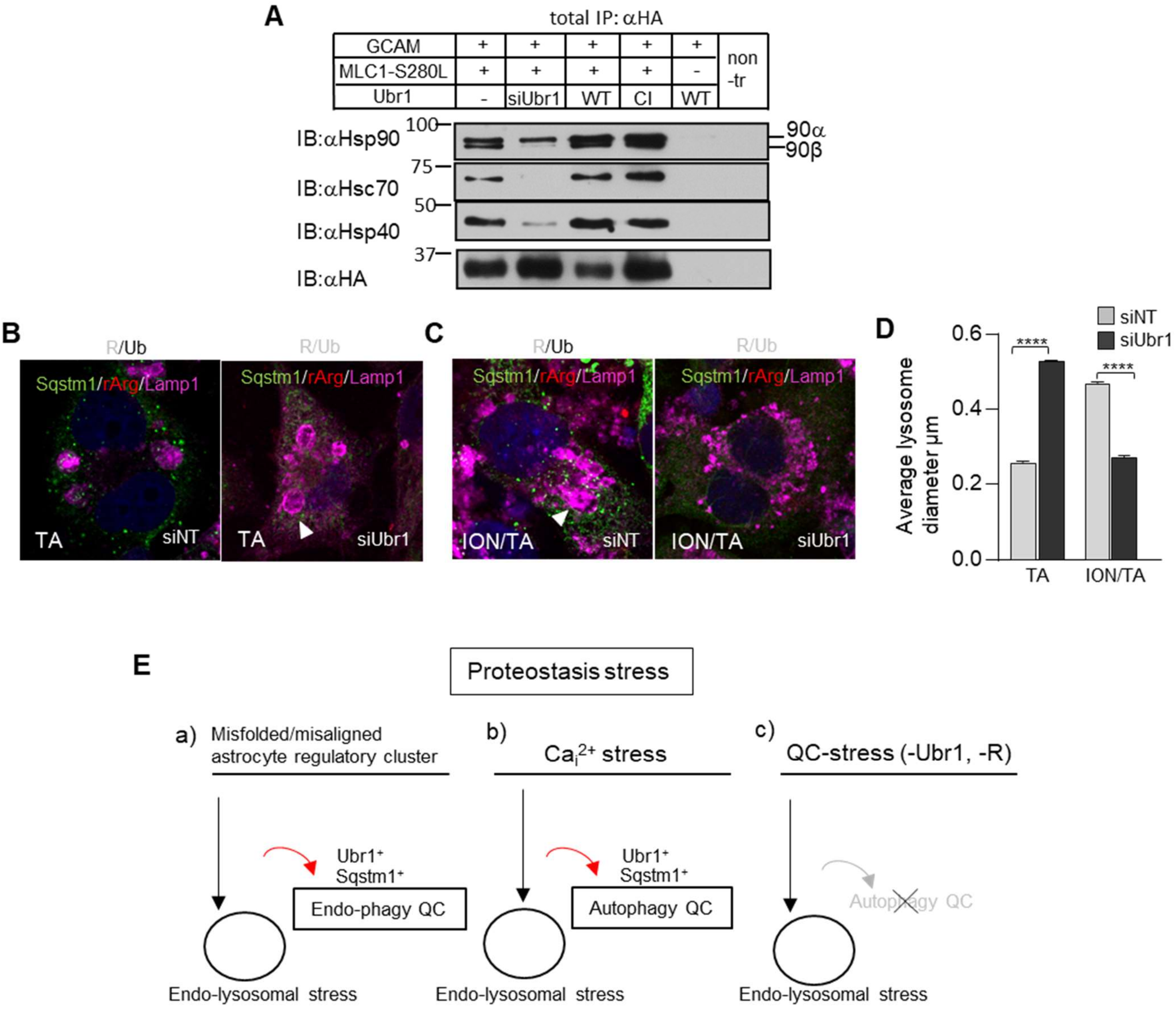

Figure S6. Ubr1 is a proteotoxic stress-induced Endo-phagy and Autophagy QC ligase

A) Association of molecular chaperones in total cell lysates with MLC1-S280L in Ubr1 (related to figure 7E). Cells were depleted for Ubr1 or overexpressed with flag-Ubr1-wt or CA. Coimmunoprecipitation was done using anti-HA and probed against indicated molecular chaperones. **B)** Ubr1 and arginylation protect against endolysosomal stress. The morphological analysis of lysosomal response to Ubr1 depletion and/or arginylation depletion by arginyltransferase 1 (Ate1) with siRNA in Hela cells. Lamp1 was a lysosomal marker. The arrowhead indicates enlarged lysosomes. Top gray labels indicate the inactivation of the arginylation (R) and/or Ubr1 (Ub). Bar 5 μm. **C)** Monitoring Ubr1-dependent Ca^{2+}-stress QC as in panel B, but ionomycin (ION) was used to induce intracellular Ca^{2+}-stress. The arrow indicates autolysosomes and arrowhead enlarged lysosomes. Bar 5 μm. **D)** Average lysosomal diameter from experimental conditions presented in B-C. n>2000 lysosomes. Means ± SEM, p-values: ****p<0.0001. **E)** Summary of Ubr1 functions during cellular proteotoxicity to fine-tune membrane organelle and PQC in Ca^{2+}-stress and disease by using endo-phagy and autophagy pathways. Ubr1 regulates the rerouting of misfolded/misaligned astrocyte regulatory protein MLC1-cluster causing endosomal compartment proteotoxicity to Endo-

phagy QC (a), and substrates during Ca^{2+}-induced proteotoxicity to Autophagy/Endo-phagy QC (b) by alternative ubiquitin/arginine-mediated and autophagosome scaffold protein SQSTM1/P62 directed pathway. Lack of functional Ubr1 and/or argininetransferase activity result in QC-stress (c) suggesting that the Ubr1/ SQSTM1/P62 pathway is an important safeguard mechanism also in physiological conditions.
